# Lactylation of Histone H3k18 and Egr1 Promotes Endothelial Glycocalyx Degradation in Sepsis‐Induced Acute Lung Injury

**DOI:** 10.1002/advs.202407064

**Published:** 2024-12-25

**Authors:** Zongqing Lu, Pu Fang, Shuai Li, Dunling Xia, Jingjing Zhang, Xianghui Wu, Jingjing Pan, Haijian Cai, Lin Fu, Gengyun Sun, Qinghai You

**Affiliations:** ^1^ Department of Respiratory and Critical Care Medicine The First Affiliated Hospital of Anhui Medical University Hefei 230022 China; ^2^ Department of Emergency Medicine First Affiliated Hospital of Anhui Medical University Hefei 230022 China; ^3^ Department of Respiratory Intensive Care Unit Anhui Chest Hospital Hefei 230022 China; ^4^ Center for Scientific Research Anhui Medical University Hefei 230032 China; ^5^ Department of Respiratory and Critical Care Medicine Second Affiliated Hospital of Anhui Medical University Hefei 230601 China

**Keywords:** EGR1, glycocalyx degradation, KAT2B, lactylation, sepsis‐induced acute lung injury

## Abstract

Circulating lactate is a critical biomarker for sepsis‐induced acute lung injury (S‐ALI) and is strongly associated with poor prognosis. However, whether elevated lactate directly promotes S‐ALI and the specific mechanism involved remain unclear. Here, this work shows that lactate causes pulmonary endothelial glycocalyx degradation and worsens ALI during sepsis. Mechanistically, lactate increases the lactylation of K18 of histone H3, which is enriched at the promoter of EGR1 and promotes its transcription, leading to upregulation of heparanase in pulmonary microvascular endothelial cells. In addition, multiple lactylation sites are identified in EGR1, and lactylation is confirmed to occur mainly at K364. K364 lactylation of EGR1 facilitates its interaction with importin‐α, in turn promoting its nuclear localization. Importantly, this work identifies KAT2B as a novel lactyltransferase whose GNAT domain directly mediates the lactylation of EGR1 during S‐ALI. In vivo, suppression of lactate production or genetic knockout of EGR1 mitigated glycocalyx degradation and ALI and improved survival outcomes in mice with polymicrobial sepsis. Therefore, this study reveals that the crosstalk between metabolic reprogramming in endothelial cells and epigenetic modifications plays a critical role in the pathological processes of S‐ALI.

## Graphical abstract

This research demonstrates that glycolysis‐derived lactate promoted an increase in H3K18la and activates EGR1 expression during S‐ALI; in addition, KAT2B induced the EGR1‐K364 lactylation and promotes the interaction between KAT2B and EGR1 resulting in its nuclear translocation. Together, these mechanisms lead to the acceleration of glycocalyx shedding by upregulating heparanase expression.

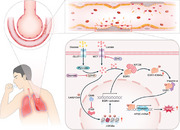

## 1 Introduction

According to the 2021 Surviving Sepsis Campaign guidelines, sepsis is defined as lethal organ dysfunction resulting from a dysregulated host response to infection, and it leads to a high mortality rate for patients in intensive care units.^[^ ^1^, ^2^ ^]^ Acute respiratory distress syndrome (ARDS) is one of the most common and severe complications arising during sepsis, with an incidence of ≈68.2% and a mortality rate of up to 35.5%,^[^ ^3^ ^]^ despite progress in a range of evidence‐based practices. One distinguishing feature of sepsis‐induced ARDS (S‐ARDS) is microcirculatory dysfunction, and another is increased endothelial permeability.^[^ ^4^ ^]^ Therefore, therapies aimed at attenuating microvascular endothelial dysfunction are essential for successfully curing S‐ARDS.

The glycocalyx, a gel‐like layer that coats the luminal surface of the vascular endothelium, is composed of multiple glycolipids, glycoproteins, proteoglycans, and adherent plasma proteins.^[^ ^5^, ^6^ ^]^ Proteoglycans are believed to be the primary constituent of the glycocalyx, with heparan sulfate proteoglycans (HSPGs) accounting for ≈50–90% of the total proteoglycan content.^[^ ^7^, ^8^ ^]^ As an endothelial gatekeeper, the glycocalyx plays crucial roles in affecting the pathological processes of S‐ALI, including but not limited to maintaining a selective permeability barrier for the pulmonary microvasculature,^[^ ^9^ ^]^ influencing blood cell–vessel wall interactions,^[^ ^8^ ^]^ and sensing and altering fluid shear stress in the microcirculation.^[^ ^10^ ^]^ Numerous preclinical and clinical studies have demonstrated that soluble markers of glycocalyx degradation are closely correlated with the severity of lung injury and the prognosis of sepsis patients.^[^ ^11^, ^12^, ^13^, ^14^ ^]^ Thus, elucidating the potential molecular mechanism of glycocalyx degradation is a crucial requirement for improving treatment modalities for S‐ALI. Previous studies have confirmed that increased enzymatic degradation that exceeds synthesis is the primary cause of glycocalyx impairment. Enzymatic degradation is carried out mainly by matrix metalloproteinases, disintegrin proteins, heparanase (HPSE), and hyaluronidases.^[^ ^5^, ^14^ ^]^ However, the mechanism driving the upregulation of these specific lyases under stress conditions such as sepsis needs further investigation.

Glucose metabolic reprogramming plays a key role in the progression of sepsis, and the primary feature of this reprogramming is increased glycolysis. Under exposure to inflammatory stimuli, almost 98% of the glucose in the vascular endothelium is metabolized into lactate, followed by the synthesis of adenosine triphosphate through aerobic glycolysis; in contrast, little glucose is routed into oxidative phosphorylation.^[^ ^15^, ^16^ ^]^ Despite the strong association between the tissue perfusion status and mortality in sepsis patients,^[^ ^1^ ^]^ lactate was initially considered only as a major metabolic substrate needed to meet the energetic requirements of cells. However, accumulating evidence has demonstrated that lactate is not only a biomarker but also an important signaling molecule that participates in endothelial dysfunction.^[^ ^17^, ^18^ ^]^ While some clinical research has indicated a potential correlation between the levels of serum lactate and HPSE, direct evidence showing the involvement of lactate in glycocalyx degradation is lacking.^[^ ^19^ ^]^

Epigenetic modifications are extensively involved in sepsis‐induced organ injury. Moreover, Zhang et al. recently reported that proteins can be directly modified by lactate through the addition of a lactyl group to lysine residues.^[^ ^20^ ^]^ This posttranslational modifications (PTM), which is termed “lactylation,” has also been verified to dynamically control gene expression and the cellular localization and function of key proteins, which in turn contributes to organ dysfunction during sepsis.^[^ ^21^, ^22^, ^23^ ^]^ However, whether lactylation is involved in the development of S‐ALI is unknown.

Here, we provided new evidence indicating that lactylation is involved in S‐ALI process. The specific mechanisms included increases in the lactate level and histone H3 lysine 18 lactylation (H3K18la) induced by sepsis, which activated EGR1/HPSE signaling, promoting the degradation of the glycocalyx barrier. Furthermore, we found that the GNAT domain of KAT2B also directly mediated K364 lactylation of EGR1, which promoted its nuclear localization. Inhibition of lactylation or silencing of EGR1 attenuated glycocalyx degradation, revealing a potential strategic target for curing S‐ALI. Our findings provided a novel perspective on the mechanism of the metabolic reprogramming‐epigenetic modifications‐organ dysfunction during sepsis.

## 2 Results

### 2.1 An Increased Lactate Level Is Corrected with a High Incidence of ARDS and Poor Prognosis in Sepsis Patients

As a biomarker for sepsis severity, the lactate level is considered an independent risk factor for poor prognosis in sepsis patients.^[^ ^24^, ^25^ ^]^ However, few studies have evaluated the associations between the blood lactate level and the incidence and severity of ARDS in sepsis patients. To determine the role of lactate in S‐ALI, we first aimed to determine the correlation between the lactate level and unfavorable clinical outcomes in patients with sepsis by an openly available clinical database called Medical Information Mart for Intensive Care‐IV (MIMIC‐IV), version 2.0. A total of 10 576 sepsis patients, including 890 ARDS patients, are represented (Figure S1A, Supporting Information). After adjusting for age, sex, comorbidities, and Sequential Organ Failure Assessment (SOFA) score, the initial lactate level was found to be an independent risk factor for the incidence of ARDS, the need for invasive mechanical ventilation (IMV), and ICU mortality in sepsis patients regardless of the infection site (Figure S1B, Supporting Information). Restricted cubic spline analysis also revealed dose‒response relationships between the lactate level and the above mentioned outcomes (Figure S1C‐E, Supporting Information). In addition, the risk of death in sepsis‐induced ARDS (S‐ARDS) patients increased sharply when the blood lactate concentration was ≥6 mmol L^−1^ (Figure S1F, Supporting Information).

The ratio of the partial pressure of oxygen in arterial blood (PaO_2_) to the fraction of inspired oxygen (PaO_2_/FiO_2_) and the alveolar‐arterial oxygen tension difference (A‐aDO_2_) are the main indicators reflecting the status of oxygenation and oxygen uptake. Our results showed clear correlations between the lactate level, PaO_2_/FiO_2_ (Figure S1G, Supporting Information), and A‐aDO_2_ (Figure S1H, Supporting Information) in all sepsis patients. Notably, compared with those in patients without ARDS, these correlations were significantly greater in patients with S‐ARDS (Figure S1I–L, Supporting Information). Additional baseline data and clinical outcomes are shown in Table S1, Supporting Information.

### 2.2 Lactate Promotes Pulmonary Vascular Permeability and ALI in Mice with Polymicrobial Sepsis

**Figure 1:**
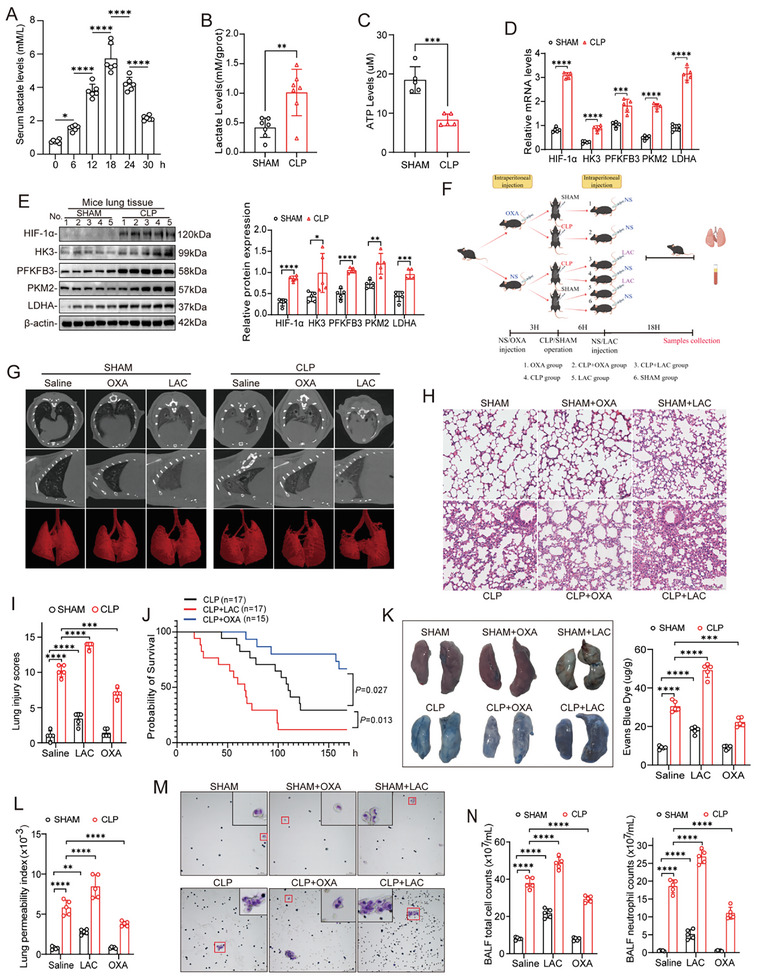
Lactate promotes ALI and pulmonary vascular permeability in mice with polymicrobial sepsis. A) Changes in serum L‐lactic acid levels in mice at various time points after CLP surgery (n = 6 mice per group). B) The lung tissue L‐lactic acid levels in mice 24 h after Sham/CLP surgery (n = 6 mice per group). C) The lung tissue ATP levels in mice 24 h after Sham/CLP surgery (n = 5 mice per group). D,E) The transcription and protein levels of the key enzymes of glycolytic pathway in lung tissue 24 h after Sham/CLP surgery (n = 5 mice per group). F) Diagram of animal experimental procedures. G) Detailed images of pulmonary inflammation were derived with Micro‐CT and 3D reconstruction, in which defect site represent the exudative lesions and consolidation. H) Haematoxylin‐eosin (HE) staining of the lung tissue sections and I) lung injury scores following CLP induced sepsis (n = 5 mice per group). Scale bar, 100 µm. J) Survival rates among CLP (n = 17), CLP+LAC (n = 17), and CLP+OXA (n = 15) group were compared by Kaplan‐Meier test. K) Evaluation of pulmonary vascular leakages using Evans blue tracer. Leakage degree was quantified by detecting the Evans blue dye contents in lung homogenate. L) Calculating the lung permeability index in each group by the following equation: protein content of bronchoalveolar lavage fluid (BALF)/protein content in the plasma (n = 5 mice per group). M) Modified Wright‐Giemsa staining of BALF precipitates. The red boxes show the neutrophils. Scale bar, 50 µm. N) The counts of total cells and neutrophils in BALF of each group. All data were represented as the means ± SD, *p < 0.05, **p < 0.01, ***p < 0.001, and ****p < 0.0001; ns, not significant.

To improve our understanding of whether lactate exacerbates ALI during sepsis, we established a sepsis model in C57BL/6 mice by performing cecal ligation and puncture (CLP) as previously described.^[^ ^26^ ^]^ As shown in **Figure** **1**A, the serum lactate concentration peaked at 18 h after CLP and decreased thereafter. We also measured the levels of lactate and ATP in lung tissue at the time of the peak serum lactate level and found that the lactate level was significantly increased while the ATP level was decreased in the CLP group compared with the sham group (Figure 1B,C). Reports have indicated that the Warburg effect is elevated and associated with immunological dysregulation and organ dysfunction during sepsis.^[^ ^27^, ^28^ ^]^ Consistent with this observation, we found that the transcription and protein expression levels of HIF‐1α, HK3, PFKFB3, PKM2, and LDHA were increased in mouse lung tissue during S‐ALI (Figure 1D,E). To explore whether lactate is involved in the pathological process of S‐ALI, we randomly assigned mice to six groups, as shown in Figure 1F, and administered sodium oxamate (OXA; 0.75 g kg^−1^ body weight) 3 h before modeling or lactate (0.5 g kg^−1^ body weight) 6 h after modeling by intraperitoneal injection.^[^ ^18^ ^]^ The results of imaging and histopathological analysis suggested that lactate supplementation further aggravated lung injury in septic mice, whereas OXA alleviated lung injury (Figure 1G‐I). It is generally accepted that under normal physiological conditions, excess lactic acid can be metabolized without affecting the body. However, we also observed a certain degree of lung injury in the sham group after lactate injection (Figure 1G‐I). As expected, compared with saline treatment, lactate administration decreased the 7‐day survival rate of septic mice by 23.53%, while pretreatment with OXA increased the survival rate by 18.1% (Figure 1J).

Substantial evidence has shown that increased vascular permeability resulting from endothelial dysfunction is a major contributor to S‐ALI.^[^ ^4^, ^18^ ^]^ To investigate whether increased serum lactate contributes to increased pulmonary vascular permeability, we injected Evans blue dye (EBD) via the tail vein and quantified its penetration into lung tissues 1 h later. We observed substantial bilateral pulmonary vascular leakage in the CLP group, as evidenced by the increased penetration of EBD into lung tissue (Figure 1K). Notably, the addition of exogenous lactate further exacerbated sepsis‐induced pulmonary vascular permeability, while pretreatment with OXA significantly attenuated this effect (Figure 1K). These findings were further confirmed by calculating the pulmonary vascular permeability index (Figure 1L). Subsequently, we counted cells in bronchoalveolar lavage fluid (BALF) and Giemsa‐stained BALF smears 24 h after CLP or sham surgery. Lactate supplementation further increased the total cell count and the neutrophil count in BALF after CLP (Figure 1M,N). However, suppression of lactate production by OXA treatment significantly reduced the abovementioned cell counts (Figure 1M,N). Together, these findings reveal that excess lactate, generated via increased aerobic glycolysis, plays an important pathological role in increasing pulmonary vascular permeability and aggravating ALI during sepsis.

### 2.3 Lactate Exacerbates Endothelial Glycocalyx Degradation during Sepsis

**Figure 2:**
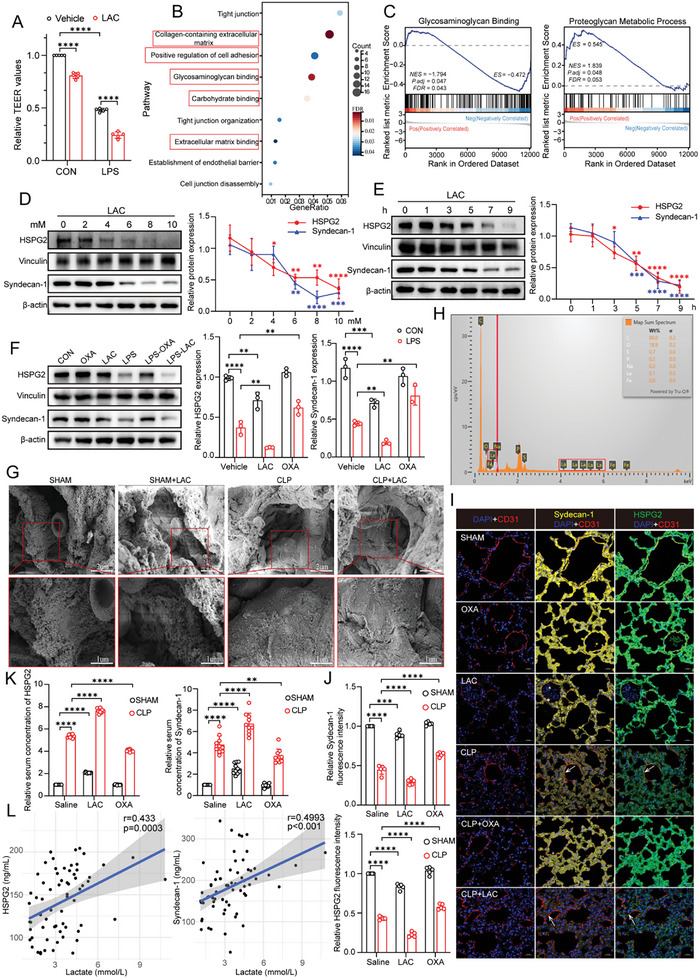
Lactate exacerbates glycocalyx degradation on MPMVECs and in vivo during sepsis. A) Effects of LPS (12 ug mL^−1^ for 12 h), lactate (8 mM for 7 h), and LPS+lactate on the trans‐endothelial electrical resistance (TEER) of MPMVECs (n = 5). B) Kyoto Encyclopedia of Genes and Genomes (KEGG) pathway analysis of differential genes based on transcriptome sequencing analysis of mRNA expressions in MPMVECs after lactate treatment. C) Gene Set Enrichment Analysis of whole transcriptome differential expression genes in MPMVECs after lactate treatment. D) The effects of different concentrations of lactate on the expression of HSPG2 and syndecan‐1 in MPMVECs (n = 3). E) The effects of different treatment times of lactate on the expression of HSPG2 and syndecan‐1 in MPMVECs (n = 3). F) MPMVECs were pre‐treated with OXA (20 mM for 3 h) or PBS, followed stimulated by LPS (12 ug mL^−1^ for 12 h), lactate (8 mM for 7 h) or both. HSPG2 and syndecan‐1 were detected by Western blot (n = 3). G) SEM imaging of the glycocalyx structure on the surface of pulmonary vascular in vivo using tracer lanthanum staining after lactate administration in Sham and CLP groups. Scale bar, 2 µm. H) Detection of the elements of the glycocalyx structure by energy‐dispersive X‐ray spectroscopy. I,J) Representative immunofluorescent staining images of HSPG2 (green) and syndecan‐1 (yellow) in endothelial surface of the lung tissues. Endothelial surface were stained with CD31 (red), and nuclei were stained with DAPI (blue). Scale bar, 20 µm; J) quantification of fluorescence intensity was analyzed by ImageJ (n = 5 per group). K) Relative serum concentration of HSPG2 and syndecan‐1 after lactate or OXA administration in Sham and CLP group (n = 10 per group). L) Correlation analysis of serum HSPG2, syndecan‐1 and blood lactate levels in S‐ARDS patients. All data were represented as the means ± SD, *p < 0.05, **p < 0.01, ***p < 0.001, and ****p < 0.0001; ns, not significant.

Our previous studies indicated that the stability of pulmonary microvascular endothelial barrier structure and function is pivotal for maintaining the balance of vascular permeability.^[^ ^29^, ^30^ ^]^ Thus, to determine whether lactate influenced the pulmonary microvascular endothelial barrier, we isolated and cultured mouse pulmonary microvascular endothelial cells (MPMVECs) in vitro and then treated the cells with different concentrations of lactate and examined their permeability. As shown in Figure S2A, Supporting Information, treatment with 8 mM lactate for 7 h markedly increased the permeability of the MPMVEC monolayer, as evidenced by the decreased transendothelial electrical resistance (TEER). In addition, 8 mM lactate exacerbated the increase in MPMVEC permeability induced by lipopolysaccharide (LPS) (**Figure** **2**A). To avoid the influence of decreased pH, we treated MPMVECs under the same acidic condition (pH 6.9) or with the same concentration of sodium lactate (NaLa). Interestingly, treatment with NaLa reduced the TEER values of the MPMVEC monolayer compared with those under the acidic condition alone (Figure S2B, Supporting Information), suggesting that lactate ions were the primary contributors to the influence of lactate on permeability.

Subsequently, we performed transcriptome sequencing of MPMVECs after lactate stimulation. Kyoto Encyclopedia of Genes and Genomes (KEGG) analysis revealed that the major pathways enriched in the differentially expressed genes were related to intercellular tight junctions, the extracellular matrix, and glycocalyx regulation (Figure 2B). In addition, gene set enrichment analysis indicated that the differentially expressed genes were associated primarily with the glycosaminoglycan binding and proteoglycan metabolic process gene sets (Figure 2C). These findings suggest that lactate may participate in extracellular glycocalyx metabolism. We subsequently evaluated changes in the glycocalyx after lactate treatment in vitro to further confirm these findings. As shown in Figure 2D,E, lactate markedly decreased the protein levels of heparan sulfate proteoglycan 2 (HSPG2) and syndecan‐1, the principal components of the glycocalyx, in a dose‐dependent and time‐dependent manner. In addition, lactate further reduced HSPG2 and syndecan‐1 levels in MPMVECs after LPS stimulation, and pre‐treatment with OXA reversed this effect (Figure 2F). This finding was also confirmed by immunofluorescence (IF) staining (Figure S2C,D, Supporting Information). LPS can increase intracellular glycolytic activity. Thus, we sought to determine whether the effect of LPS on glycocalyx degradation is partially attributable to the increased production of intracellular lactate. We pretreated MPMVECs with rotenone to block the electron transport chain involved in oxidative phosphorylation, and the same trend persisted (Figure S2E, Supporting Information). We then knocked down LDHA by siRNA and found that siRNA‐LDHA partially weakened the effect of LPS on HSPG2 and syndecan‐1 levels compared with those in the siRNA‐NC group (Figure S2F, Supporting Information). The above results demonstrated that both endogenous and exogenous lactate accumulation contributed to the degradation of the extracellular glycocalyx.

In subsequent in vivo experiments, scanning electron microscopy (SEM) and energy‐dispersive X‐ray spectroscopy (EDS) (Figure 2G,H) and IF staining (Figure 2I,J) consistently showed apparent glycocalyx shedding on the surface of the pulmonary vasculature after CLP‐mediated induction of sepsis, and lactate supplementation exacerbated this shedding. Western blot (WB) analysis and enzyme‐linked immunosorbent assay (ELISA) kits were used to measure the expression of HSPG2 and syndecan‐1 in whole lung tissue and their concentrations in serum, respectively. As shown in Figure S2G,H, Supporting Information, glycocalyx degradation accompanied disease progression in sepsis, significant differences began to appear after 12 h. Additionally, lactate supplementation further exacerbated CLP‐induced damage to the lung microvascular surface glycocalyx, while sodium OXA treatment effectively alleviated this effect (Figures S2K and S2I, Supporting Information). In summary, these data indicate that lactate promotes the degradation of the glycocalyx during the pathological process of sepsis, thereby further resulting in dysfunction of the pulmonary microvascular endothelial barrier.

For clinical verification of these findings, we examined the levels of HSPG2 and syndecan‐1 in the serum of S‐ARDS patients. Consistent with previous researches,^[^ ^13^, ^31^ ^]^ patients with S‐ARDS had significantly increased serum HSPG2 and syndecan‐1 levels compared with those in healthy donors (Figure S2J, Supporting Information), and these levels increased with ARDS exacerbation (Figure S2K, Supporting Information). In addition, the serum levels of both HSPG2 and syndecan‐1 were correlated with the oxygenation index and invasive ventilation time but not with the SOFA and Acute Physiology and Chronic Health Evaluation II scores (Figure S2L–O, Supporting Information). After adjustment for age and sex, multivariate analysis revealed that the serum levels of HSPG2 and syndecan‐1 were independent risk factors for 28‐day mortality in S‐ARDS patients and that these associations were more pronounced in patients with septic shock (Figure S2P, Supporting Information). Interestingly, we also found that the serum levels of shed glycocalyx components were significantly positively correlated with the serum lactate level in S‐ARDS patients (Figure 2L). This result was consistent with the in vivo findings. The clinical information of all patients included in the analysis is shown in Table S2, Supporting Information.

### 2.4 Lactate Promotes Glycocalyx Degradation by Increasing the Expression of Heparanase

**Figure 3:**
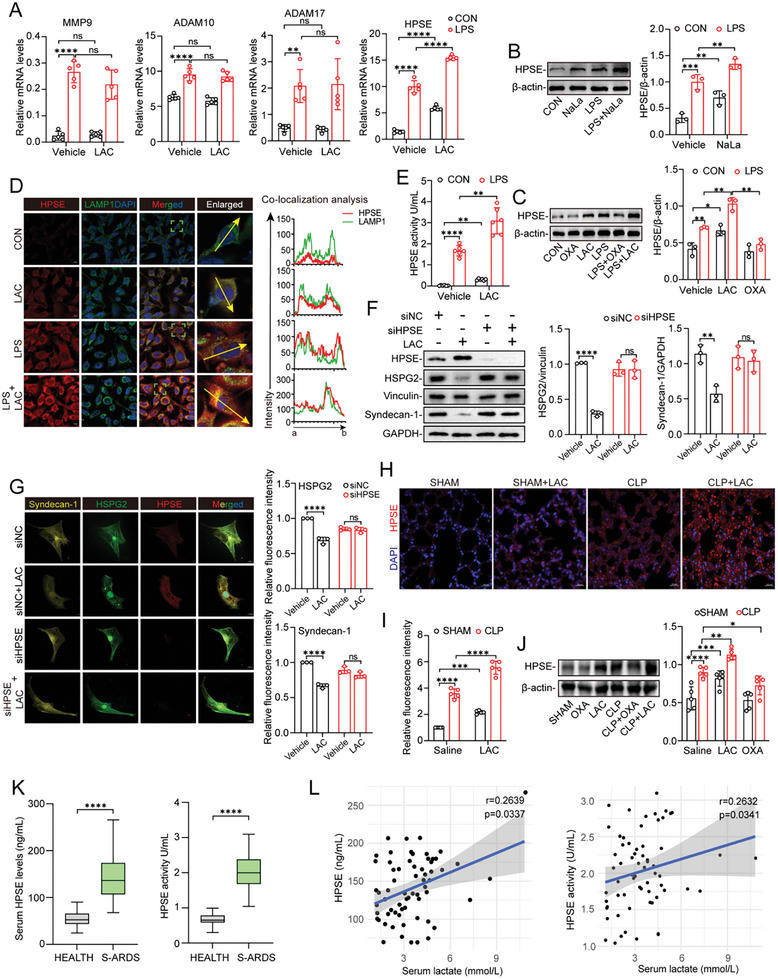
Lactate promotes glycocalyx degradation by increasing the expression of HPSE. A) RT‐qPCR was used to detect the MMP9, ADAM10, ADAM17, and HPSE mRNA level of MPMVECs after lactate (8 mM for 7 h) treatment, respectively (n = 5). B) The effect of NaLa (8 mM for 7 h) on the expression of HPSE in MPMVECs (n = 3). C) MPMVECs were pre‐treated with OXA (20 mM for 3 h) or PBS, followed stimulated by LPS (12 ug mL^−1^ for 12 h), lactate (8 mM for 7 h) or both. HPSE was detected by Western blot (n = 3). D) MPMVECs were treated with LPS (12 ug mL^−1^ for 12 h), lactate (8 mM for 7 h), or both. HPSE (red) and LAMP1 (green) co‐localization was examined by confocal microscope (scale bar, 10um). Nucleus was indicated by DAPI (blue) staining. Co‐localization analysis was performed by ImageJ. Scale bar, 10 µm. E) The levels of HPSE activity in MPMVECs after lactate treatment (8 mM for 7 h) (n = 6). F) MPMVECs were transfected with siRNAs for HPSE and scramble control siRNA before 8 mM lactate stimulation for 7 h, the expression levels of HSPG2, syndecan‐1, and HPSE were detected by Western blot (n = 3). G) HSPG2 (green), syndecan‐1 (yellow), and HPSE (red) immunofluorescence staining in the MPMVECs transfected with si‐NC or si‐HPSE after lactate (8 mM for 7 h) stimulation. Scale bar, 10 µm (n = 3). H) Immunofluorescence and I) relative intensity quantification of HPSE of mice lung tissue sections. Scale bar, 20 µm (n = 5). J) The expression levels of HPSE in lung tissue in different groups (n = 5). K) Comparation of serum HPSE concentration and activity between health individuals and S‐ARDS patients. L) Correlation analysis of serum HPSE concentration and activity with blood lactate levels in S‐ARDS patients. All data were represented as the means±SD, *p < 0.05, **p < 0.01, ***p < 0.001, and ****p < 0.0001; ns, not significant.

To further investigate the mechanism underlying glycocalyx degradation, we first examined the effect of lactate on cell viability, death, and apoptosis. Notably, varying the concentration of lactate within 12 mM did not lead to a substantial decrease in cell viability (Figure S3A, Supporting Information), and further flow cytometric analysis also revealed that 8 mM lactate supplementation did not significantly induce MPMVEC death or apoptosis (Figure S3B, Supporting Information). Subsequently, we further investigated the impact of lactate on glycocalyx synthesis. RT‒qPCR analysis confirmed that lactate did not alter the transcript level of HSPG2 or syndecan‐1 (Figure S3C, Supporting Information). As proteases secreted from endothelial cells play important roles in glycocalyx degradation under exposure to inflammatory stimuli,^[^ ^5^ ^]^ we measured the expression of a series of specific proteases in MPMVECs following lactate treatment. Lactate exhibited the most dramatic promoting effect on the mRNA expression of HPSE among these proteases (**Figure** **3**A) and promoted HPSE protein expression in a time‐ and concentration‐dependent manner (Figure S3D, Supporting Information). We then treated MPMVECs with NaLa under the same experimental condition (8 mM for 7 h) and also found increases in HPSE transcription and protein expression (Figure S3E,B, Supporting Information). Similarly, LPS upregulated the protein expression of HPSE. Lactate further enhanced the LPS‐induced increase in HPSE expression, while OXA exerted an inhibitory effect (Figure 3C). It has been reported that biologically active HPSE is stored in lysosomes after proteolytic enzyme treatment.^[^ ^32^ ^]^ IF staining with anti‐HPSE and anti‐Lamp1 antibodies showed that lactate treatment strongly increased the cytoplasmic level of HPSE in mouse PMVECs and increased the colocalization of HPSE with lysosomes (Figure 3D). Consistent with these results, increased HPSE activity was also detected by ELISA in whole‐cell lysates after lactate treatment (Figure 3E). Interestingly, we similarly observed that HPSE activity was increased by NaLa treatment (Figure S3F,G, Supporting Information). To investigate whether excess lactate promotes accelerated glycocalyx degradation by facilitating HPSE expression, we transfected MPMVECs with negative control small interfering RNA (siRNA‐NC) or siRNA‐HPSE and evaluated HSPG2 and syndecan‐1 protein expression after lactate treatment by WB analysis and IF staining. As shown in Figure 3F,G, silencing HPSE significantly attenuated the effect of lactate on HSPG2 and syndecan‐1 expression.

In vivo, IF staining of mouse lung tissue sections revealed that polymicrobial sepsis increased the HPSE level in lung tissues and that this increase was further promoted by lactate administration (Figure 3H,I). WB analysis of tissue lysates also revealed a similar trend (Figure S3H, Supporting Information). Conversely, inhibition of lactate production by OXA treatment attenuated the CLP‐induced increase in HPSE expression in lung tissue (Figure 3J). Notably, stimulation with lactate alone increased HPSE expression in lung tissue also in the sham group (Figure 3H,J).

Next, we examined the level and activity of HPSE in the serum of S‐ARDS patients. In agreement with the findings of a previous study,^[^ ^19^ ^]^ the serum HPSE content and activity were significantly greater in S‐ARDS patients (Figure 3K) and increased with increasing disease severity (Figure S3I, Supporting Information). Moreover, both the content and activity of serum HPSE were negatively correlated with the oxygenation index in S‐ARDS patients (Figure S3J, Supporting Information). Notably, the initial blood lactate level was also showed modest correlations with serum HPSE content and activity (Figure 3L). Collectively, these data suggest that increased expression and activity of HPSE mediate the lactate‐induced exacerbation of glycocalyx degradation on the surface of pulmonary microvessels during sepsis.

### 2.5 Global Lactylation and H3K18la Levels are Increased During Sepsis

**Figure 4:**
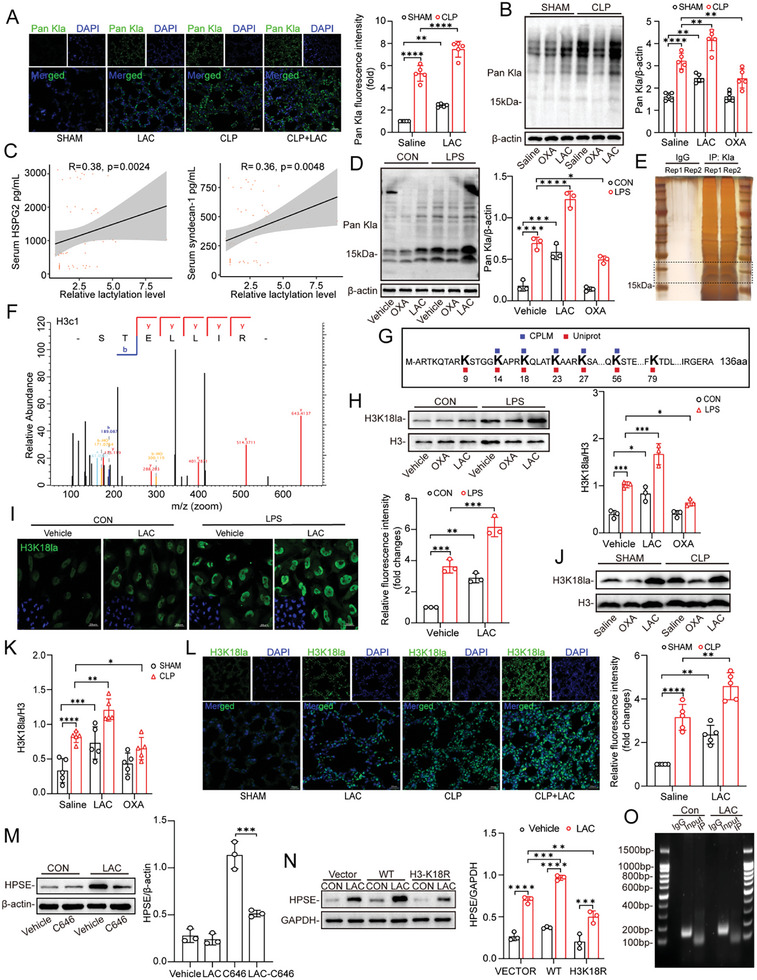
Global lactylation and H3K18la levels in the lung are increased during sepsis. A) Representative immunofluorescent staining images of Pan Kla (green) of the mice lung tissue sections and the nuclei were stained with DAPI (blue), Scale bar, 20 µm. Quantification of fluorescence intensity was analyzed by ImageJ (n = 5). B) The Pan Kla immunoblots of lung tissue in different groups (n = 5). C) Correlation analysis of serum HSPG2 and syndecan‐1 concentrations with relative lactylation levels in lung tissue. Relative lactylation levels were measured by Western blot. D) The Pan Kla immunoblots of MPMVECs (n = 3). E) Silver stained SDS‐PAGE of lactylated proteins in MPMVECs after immunoprecipitation assay with anti‐Pan Kla antibody. F) Silver staining‐mass spectrometry (MS) of the band between 15–25 kDa in SDS‐PAGE. G) Lactylation sites on Histone H3 were predicted on CPLM (http://cplm.biocuckoo.cn/index.php) and Uniprot (https://www.uniprot.org/). H) The protein levels of H3K18la in MPMVECs after pre‐treated with OXA (20 mM for 3 h) or PBS, followed stimulated by LPS (12 ug mL^−1^ for 12 h), lactate (8 mM for 7 h) or both (n = 3). I) Immunofluorescence and relative intensity quantification of H3K18la (green) in MPMVECs after treatment with LPS (12 ug mL^−1^ for 12 h), lactate (8 mM for 7 h), or both. Scale bar, 20 µm (n = 3). J,K) Expression levels of H3K18la in mice lung tissue of different groups (n = 5). L) Immunofluorescence and relative intensity quantification of H3K18la (green) of mice lung tissue sections in different groups. Scale bar, 20 µm (n = 5). M) The effect of pre‐treated C646 (5uM) for 3 h on the protein expression of HPSE in MPMVECs (n = 3). N) Expression levels of HPSE in control vector, H3‐WT, and H3‐K18R overexpressed MPMVECs after lactate stimulation or not (n = 3). O) ChIP‐PCR assay were used to detect the enrichment of H3K18la at the promoter region of HPSE after lactate stimulation (8 mM) or not. All data were represented as the means±SD, *p < 0.05, **p < 0.01, ***p < 0.001, and ****p < 0.0001; ns, not significant.

To explore the potential mechanisms responsible for the increase in HPSE expression induced by lactate in PMVECs, we first used actinomycin D and cycloheximide to monitor HSPE mRNA and protein stability, respectively, in the presence and absence of lactate. As shown in Figure S4A,B, Supporting Information, lactate did not alter the mRNA or protein stability of HPSE, indicating that the lactate‐induced increase in the HPSE level in PMVECs is due to an increase in HPSE expression at the transcriptional level. Lactylation has been confirmed to regulate the function of the modified proteins, and it plays an important role in numerous disease processes.^[^ ^21^, ^23^ ^]^ However, the exact function of lactylation in S‐ALI remains unclear. We then examined the lactylation levels in the lung tissue of mice with sepsis using an anti‐pan‐lactyl‐lysine (Pan Kla) antibody. The results demonstrated that, the global lactylation level in mouse lung tissue was elevated after CLP (Figure S4C, Supporting Information) and was significantly affected by the serum lactate level (**Figure** **4**A,B). In addition, we detected significant correlations between the lung lactylation level and the serum HSPG2 and syndecan‐1 levels (Figure 4C). These results indicate that lactylation may be involved in glycocalyx degradation.

Subsequently, we treated MPMVECs with exogenous lactate and measured the global lactylation level. The lactylation level but not the acetylation level increased continuously with lactate stimulation time and concentration (Figure S4D–G, Supporting Information). Moreover, lactate supplementation further increased LPS‐induced lactylation levels, while OXA attenuated LPS‐induced lactylation (Figure 4D and Figure S4H, Supporting Information). Since the lactylated proteins were localized mainly in the nucleus according to IF staining and the predominant band visible on the WB corresponded to a molecular weight of 15–20 kDa, we then immunoprecipitated the lactylated proteins using the anti‐Pan Kla antibody and performed sodium dodecyl sulfate–polyacrylamide gel electrophoresis with silver staining followed by mass spectrometry (MS). The results showed that the above mentioned band corresponded to histone H3 (Figure 4E,F, Table S3, Supporting Information). Then, the Compendium of Protein Lysine Modifications (CPLM) (http://cplm.biocuckoo.cn/index.php) resource and UniProt (https://www.uniprot.org/) were used to predict the lactylation sites on mouse histone H3. With both CPLM and UniProt, we identified high‐confidence sites at lysine 14 (K14), K18, K23, K27, and K56 of histone H3 (Figure 4G and Figure S4I, Supporting Information). Extensive work previously demonstrated that histone H3 K18 lactylation (H3K18la) directly participates in the transcriptional regulation of specific genes.^[^ ^33^, ^34^ ^]^ Hence, we focused our subsequent study on H3K18la. The H3K18la level but not the H3K18ac level showed the same trend as the global lactylation level in MPMVECs after exogenous lactate treatment (Figure S4J,K, Supporting Information). Notably, lactate treatment also markedly up‐regulated the protein level of P300, a key histone acetyltransferase that regulates histone lactylation (Figure S4L, Supporting Information). Similarly, WB analysis and IF staining indicated that alterations in the intracellular lactate level induced by LPS or OXA treatment also effectively influenced the level of H3K18la in MPMVECs (Figure 4H,I). Furthermore, the H3K18la level was significantly increased in lung tissue after CLP (Figure S4M, Supporting Information) and was further increased by injection of additional lactate (Figure 4J–L).

To investigate whether lactate affects HPSE gene expression through an increase in the H3K18la level, MPMVECs were treated with 25 µM C646 for 24 h prior to lactate stimulation to inhibit P300 activity. Notably, the stimulatory effect of lactate on HPSE expression was considerably impaired by C646 (Figure 4M). Next, we constructed lentiviral plasmids expressing wild‐type H3 (H3‐WT) and an H3 mutant with a point mutation resulting in the substitution of K18 with arginine (H3‐K18R). The mutation was verified by Sanger sequencing and WB assay (Figure S4N,O, Supporting Information). Notably, the expression of the H3‐K18R mutant significantly reduced HPSE expression after lactate treatment compared to that in the H3‐WT group (Figure 4N). Furthermore, IF staining revealed that H3‐K18R significantly attenuated lactate‐induced cell surface glycocalyx degradation compared with that in the H3‐WT group (Figure S4P, Supporting Information). Finally, we examined the binding of H3K18la to the HPSE promoter region. However, the chromatin immunoprecipitation–PCR (ChIP–PCR) results indicated that H3K18la does not directly bind to the HPSE promoter region (Figure 4O). Collectively, these results suggest that H3K18la participates in lactate‐induced glycocalyx degradation through indirect regulation of HPSE expression.

### 2.6 H3K18la Upregulates the Expression of HPSE by Promoting the Activation of EGR1

**Figure 5:**
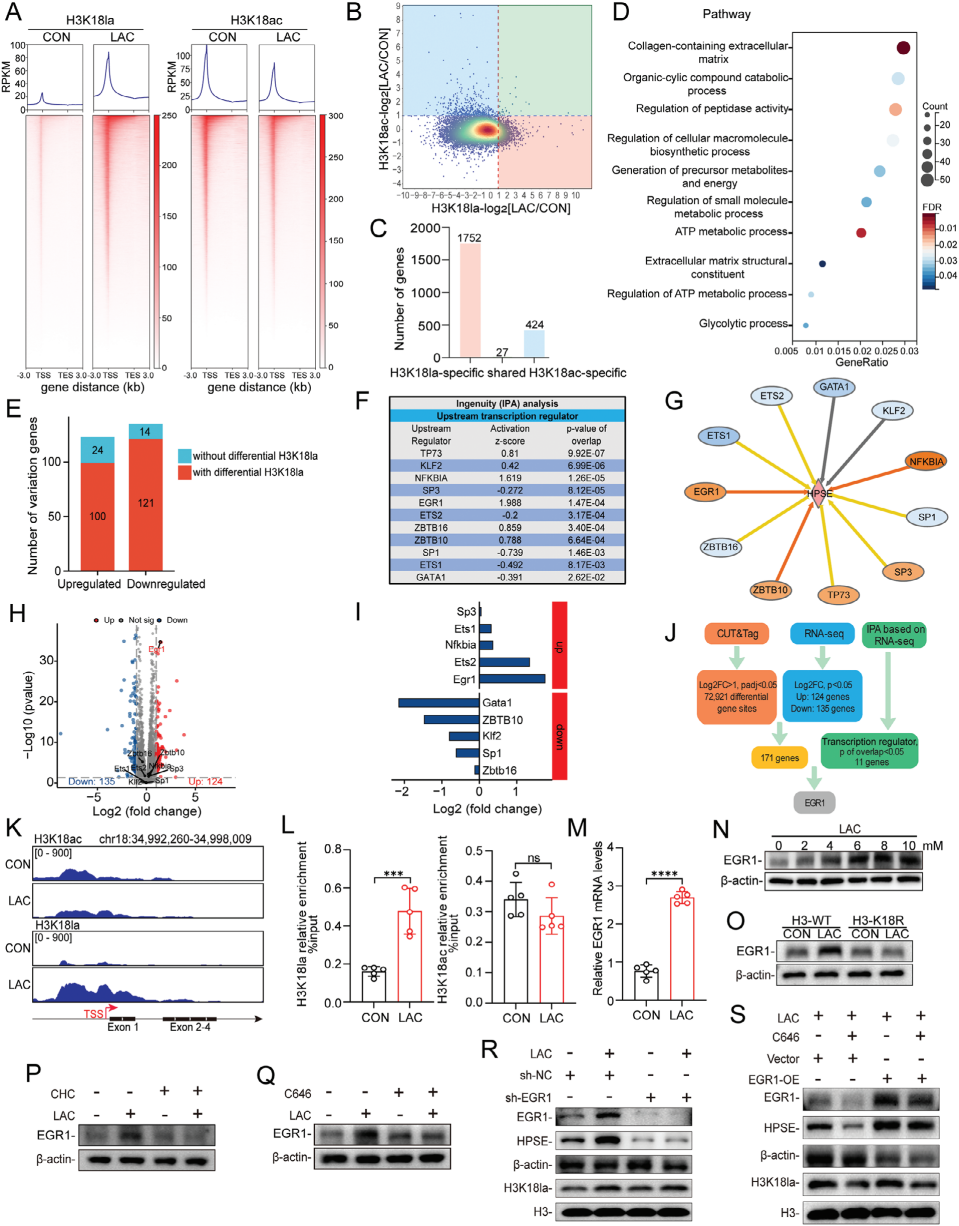
H3K18la enhances the expression of HPSE by increasing the activation of EGR1. A) Heatmaps for genomic occupancy of H3K18la and H3K18ac±3kb flanking transcription start site in MPMVECs after be stimulated with 8 mM lactate for 7h. Color depth indicates the relative number of reads, genes with similar distribution patterns are clustered together through a clustering algorithm to show the binding trends of lactylation modifications on all genes. B) Scatterplot and C) bar plot showing genes with promoters marked by increases in only H3K18la (H3K18la log_2_[LAC/CON] ≧ 1 and H3K18ac log_2_[LAC/CON] < 1; H3K18la‐specific genes), increases in both H3K18la and H3K18ac (H3K18la log_2_[LAC/CON] ≧ 1 and H3K18ac log_2_[LAC/CON] ≧ 1; shared genes), or increases in only H3K18ac (H3K18la log_2_[LAC/CON] < 1 and H3K18ac log_2_[LAC/CON] ≧ 1; H3K18ac‐specific genes). D) KEGG pathway analysis of genes bound by H3K18la in MPMVECs. E) Bar graph indicating the number of up‐regulated and down‐regulated genes with or without differential H3K18la modification. F) Potential transcription factors of HPSE by ingenuity pathway analysis based on RNA‐seq. G) An upstream regulatory network diagram shows the interactions between HPSE and its directly related up‐stream transcription factors. The different colors in the ellipses indicate the expression of genes in the transcriptome. Red ellipses indicate up‐regulated genes, and green ellipses indicate downregulated genes. The orange and gray line indicate the expression state with consistent activation or suppression between the upstream regulator and the gene, respectively, while the yellow line indicates the expression state with inconsistent activation between the upstream regulator and the gene. H) Volcano plot shows the distribution of the potential up‐stream transcription factors of HPSE in MPMVECs after lactate treatment. I) Rank of the potential up‐stream transcription factors of HPSE based on their H3K18la modifications degree. J) Bioinformatics analysis filtered EGR1 as a downstream target of H3K18la. K) IGV tracks for EGR1 from CUT&Tag analysis. L) ChIP‐qPCR assays of H3K18la and H3K18ac occupancy rates in the promoter region of EGR1 after lactate treatment (n = 5). M) RT‐qPCR was used to detect the EGR1 mRNA level in MPMVECs after lactate (8 mM for 7 h) treatment (n = 5). N) The effect of different concentrations of lactate on the EGR1 expression in MPMVECs. O) Expression levels of EGR1 in the K18R site mutation or wild‐type histone H3 overexpressed MPMVECs after 8 mM lactate treatment for 7h. P) MPMVECs were pre‐treated with 3 mM CHC for 1 h, and further stimulated with 8 mM lactate for 7h. The expression of EGR1 in cells was detected by Western blot. Q) MPMVECs were pre‐treated with 5uM C646 for 3 h, and further stimulated with 8 mM lactate for 7h. The expression of EGR1 in cells was detected by Western blot. R) Western blot assay showed that EGR1 knockdown reversed the up‐regulation of HPSE induced by increased H3K18la after 8 mM lactate treatment. S) Overexpression of EGR1 attenuated the effect of C646 on HPSE expression. All data were represented as the means±SD, *p < 0.05, **p < 0.01, ***p < 0.001, and ****p < 0.0001; ns, not significant.

To explore the mechanism by which H3K18la regulates HPSE expression, we first screened for candidate target genes involved in histone lactylation in MPMVECs via cleavage under targets and tagmentation (CUT&Tag) using anti‐H3K18la or anti‐H3K18ac antibodies. The binding region of H3K18la but not that of H3K18ac was significantly affected by lactate treatment (**Figure** **5**A). Furthermore, H3K18la was enriched primarily in the promoter and upstream regions of genes after stimulation with lactate supplementation (Figure S5A,B, Supporting Information). Most modified genes exhibited an increase in either H3K18la or H3K18ac but not both, and the H3K18la‐modified genes were significantly distinct from the H3K18ac‐modified genes in MPMVECs after lactate treatment. Only 1.2% of the modified genes (27 of 2203) exhibited significant increases in both H3K18la and H3K18ac, while up to 98% (1752 of 1779) of the H3K18la‐modified genes did not display a significant increase in H3K18ac (Figure 5B,C). KEGG enrichment analysis revealed that H3K18la‐modified genes were involved primarily in extracellular matrix regulation and cellular substance and energy metabolism (Figure 5D).

To identify H3K18la‐targeted genes that regulate HPSE expression in MPMVECs, we next combined the CUT&Tag and RNA‐seq data to classify genes into four categories according to their expression and H3K18la modification status: up‐regulated genes with or without differential H3K18la and down‐regulated genes with or without differential H3K18la (Figure 5E). Figure S5C shows the correlation between genes with increased transcription levels and enriched H3K18la in the promoter region. Furthermore, we searched for upstream regulators by using Ingenuity Pathway Analysis based on the RNA‐seq data. The results demonstrated that a total of 11 regulators may participate in regulating HPSE expression (Figure 5F,G). We found that EGR1, a key upstream transcription factor (TF) of HPSE, exhibited the most significant increases in transcription and H3K18la modification (Figure 5H,I). We then used JASPAR to predict the EGR1 binding sites within the HPSE promoter (Figure S5D, Supporting Information). Considering the predictions, we focused on EGR1 as a potential target gene that mediates the regulation of HPSE by H3K18la (Figure 5J). Integrative Genomics Viewer was used to visualize the binding sites of H3K18ac and H3K18la in the EGR1 sequence (Figure 5K). Then, to validate the CUT&Tag and RNA‐seq results, we conducted ChIP‒qPCR and RT‒qPCR analyses and confirmed H3K18la enrichment in the promoter region of EGR1 (Figure 5L) and the increased transcript and protein levels of EGR1 after lactate treatment (Figure 5M,N), respectively. However, lactate‐induced upregulation of EGR1 was significantly suppressed in cells expressing the H3‐K18R mutant (Figure 5O). A similar effect was observed when CHC was used to inhibit MCT1 expression or C646 was used to inhibit P300 expression (Figure 5P,Q). Finally, we investigated the effects of EGR1 overexpression and knockdown on the HPSE expression level in MPMVECs. EGR1 knockdown significantly inhibited the upregulation of HPSE induced by increased H3K18la (Figure 5R). Conversely, overexpression of EGR1 antagonized the C646‐induced decrease in HPSE expression (Figure 5S). In summary, these results suggest that H3K18la facilitates HPSE expression by promoting the transcription of EGR1 in MPMVECs after lactate stimulation.

### 2.7 Lactate Promotes Binding of EGR1 to Importin‐α via K364la, in turn Facilitates EGR1 Nuclear Localization

**Figure 6:**
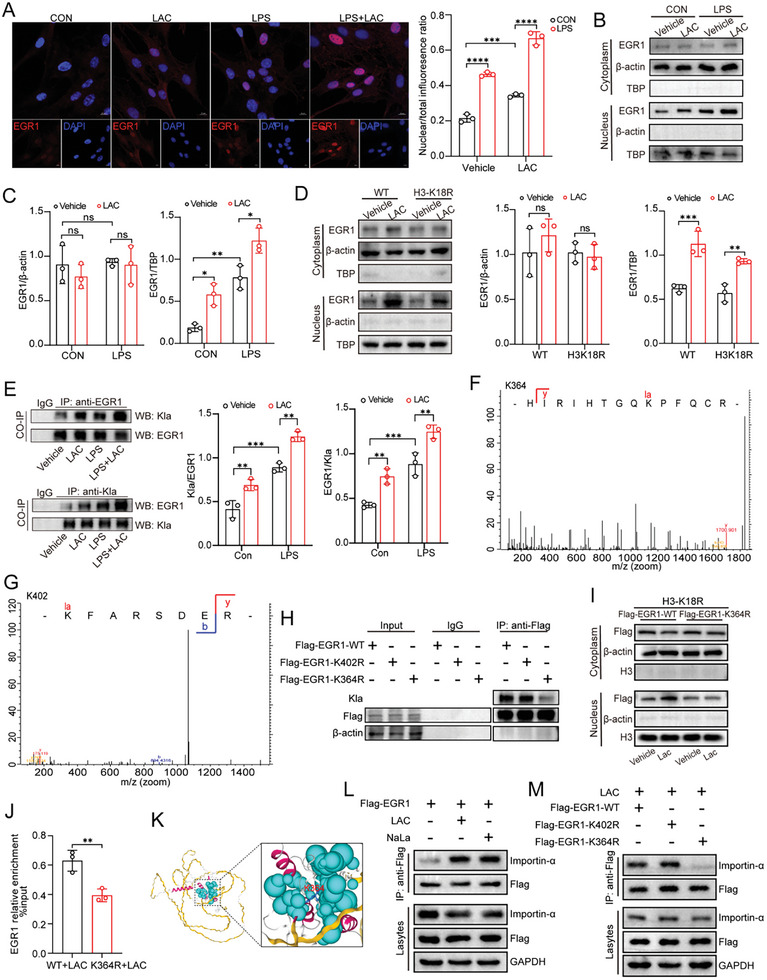
Lactate increases EGR1 binding to Importin‐α via K364la, in turn facilitating EGR1 nuclear localization A) Immunofluorescence of EGR1 (red) in MPMVECs after treatment with LPS (12 ug mL^−1^ for 12 h), lactate (8 mM for 7 h), or both. Nuclei were stained with DAPI (blue). Scale bar, 10 µm (n = 3). B‐C) After MPMVECs be treated with LPS (12 ug mL^−1^ for 12 h), lactate (8 mM for 7 h), or both, the cytoplasmic and nuclear EGR1 expression was measured by Western blot (n = 3). D) Cytoplasmic and nuclear EGR1 expression was measured in MPMVECs expressing H3‐WT or the K18R mutant after lactate (8 mM for 7 h) stimulation by Western blot (n = 3). E) Immunoprecipitation (IP) was performed to examine lactylation of EGR1 in MPMVECs after treatment with LPS (12 ug mL^−1^ for 12 h), lactate (8 mM for 7 h), or both (n = 3). F,G) Illustration of possible lactylation sites of EGR1 in the MPMVECs analyzed via IP‐LC‐MS/MS. Two possible lactylation sites of EGR1 observed are shown. H) K364R, K402R, or wild‐type Flag‐EGR1 overexpressed MPMVECs were constructed respectively through overexpression plasmid. Flag‐EGR1 proteins were pulled down by Flag antibody and detected with anti‐Pan Kla antibody. I) The effect of lactate treatment (8 mM for 7 h) on Flag‐EGR1 protein distribution after overexpressed Flag‐EGR1‐WT and Flag‐EGR1‐K364R in cells expressing the H3‐K18R mutant. J) ChIP‐qPCR assays of EGR1 occupancy rates in the promoter region of HPSE after K364R mutation (n = 3). K) Ribbon diagram of the crystal structure of mouse EGR1 protein (PDB entry P08046) was obtained from AlphaFold Protein Structure Database (https://alphafold.ebi.ac.uk). We used YINFO online platform (https://cloud.yinfotek.com) to visualize the C2H2 zinc finger domains (red) and label the K364 site (blue). L) After treatment with lactate (8 mM for 7 h) or NaLa lactate (8 mM for 7 h), co‐IP was performed to examine the interaction between Flag‐EGR1 protein and Importin‐α in MPMVECs overexpressing the Flag‐EGR1. M) After treatment with lactate (8 mM for 7 h), co‐IP was performed to examine the interaction between Flag‐EGR1 protein and Importin‐α in MPMVECs overexpressing the K364R, K402R, or wild‐type Flag‐EGR1. All data were represented as the means±SD, *p < 0.05, **p < 0.01, ***p < 0.001, and ****p < 0.0001; ns, not significant.

After being biosynthesized and processed, the nuclear translocation of TFs is tightly regulated. Several PTMs, for example, SUMOylation, have been shown to function in altering the subcellular localization of modified proteins.^[^ ^35^ ^]^ Moreover, importantly, a recent study verified that lactate can alter the nuclear localization of TFs by directly promoting their lactylation_._^[^ ^36^ ^]^ We noticed that not only was EGR1 expression affected, EGR1 was translocated more to the nucleus after lactate treatment in MPMVECs (**Figure** **6**A,C). Hence, we sought to determine whether lactate can also modulate the localization of EGR1 through its lactylation. To exclude as much as possible the influence of increased H3K18la on EGR1 upregulation, we first carried out a nuclear–cytoplasmic fractionation assay on MPMVECs expressing H3‐WT or the K18R mutant. Next, MPMVECs were transfected with the Flag‐EGR1 plasmid for exogenous overexpression of EGR1. Subsequent results showed that lactate treatment dramatically increased the nuclear localization of both exogenous and endogenous EGR1 (Figure 6D and Figure S6A, Supporting Information).

To determine whether EGR1 can be lactylated, we treated MPMVECs with lactate, LPS, or both and then disrupted protein‒protein interactions using sodium dodecyl sulfate to prevent indirect modification. We subsequently performed IP with anti‐EGR1 and anti‐Kla antibodies. The results indicated that EGR1 could be lactylated and that its lactylation was further increased after treatment with lactate or LPS (Figure 6E). Next, we identified the potential lactylation sites of EGR1 in lactate‐induced MPMVECs, which included K364 and K402, through liquid chromatography–tandem mass spectrometry (LC‒MS/MS) analysis (Figure 6F,G). As shown in Figure S6B, Supporting Information, both K364 and K402 of EGR1 were relatively conserved across species. K364R and K402R site mutants or WT‐EGR1 were constructed through the introduction of mutant plasmids containing a Flag‐tag (Figure S6C, Supporting Information). We then used an anti‐Flag antibody to pull down EGR1 and determine its lactylation level. The K364R mutation significantly reduced but the K402R mutation did not significantly affect EGR1 lactylation (Figure 6H). These findings suggested that K364 is the primary lactylation site in EGR1.

To further determine whether increased K364la of EGR1 is responsible for the lactate‐induced nuclear localization of EGR1 observed in MPMVECs, we separately over‐expressed Flag‐EGR1‐WT and Flag‐EGR1‐K364R in cells expressing the H3‐K18R mutant. The results presented in Figure 6I demonstrate a significant decrease in the nuclear localization of Flag‐EGR1 in EGR‐K364R cells compared to EGR1‐WT cells. Furthermore, IF staining revealed that after K364R mutation, the nuclear translocation of EGR1 and its colocalization with KLA in the nucleus were significantly reduced (Figure S6D, Supporting Information). In addition, the K364R mutation decreased the binding of EGR1 to the promoter region of HPSE and the transcript level of HPSE after lactate treatment, as determined by ChIP‒qPCR and RT‒qPCR analyses (Figure 6J and Figure S6E, Supporting Information).

TFs contain a requisite nuclear localization signal (NLS) that can be recognized by carrier proteins, such as importins, also called karyopherins, for translocation through the nuclear pore. Generally, NLSs consist of consecutive basic amino acid residues.^[^ ^37^ ^]^ Cys2‐His2‐type (C2H2) zinc finger domains (ZFDs) can also serve as NLSs to facilitate the nuclear entry of specific zinc finger proteins that contain a triple C2H2 ZFD or multiple adjacent C2H2 ZFDs.^[^ ^38^ ^]^ In addition, a recent study revealed that basic amino acid residue mutations in ZFDs can influence TFs binding to importin‐α, which in turn alters its nuclear transport activity.^[^ ^39^ ^]^ Interestingly, in the tertiary structure of EGR1, K364 was located in one of the three C2H2 ZFDs (Figure 6K). Therefore, we examined the ability of EGR1 to bind to importin‐α in MPMVECs following lactate or NaLa stimulation and found that treatment with either lactate or NaLa facilitated the binding of EGR1 to importin‐α (Figure 6L). However, compared with expression of EGR1‐WT or the EGR1‐K402R mutant, expression of the EGR1‐K364R mutant strongly reduced this binding (Figure 6M). Overall, these results indicate that lactate promotes the binding of EGR1 to importin‐α through an increase in EGR1 K364la, thereby facilitating the nuclear localization of EGR1.

### 2.8 Identification of KAT2B as an EGR1 Lactyltransferase

**Figure 7:**
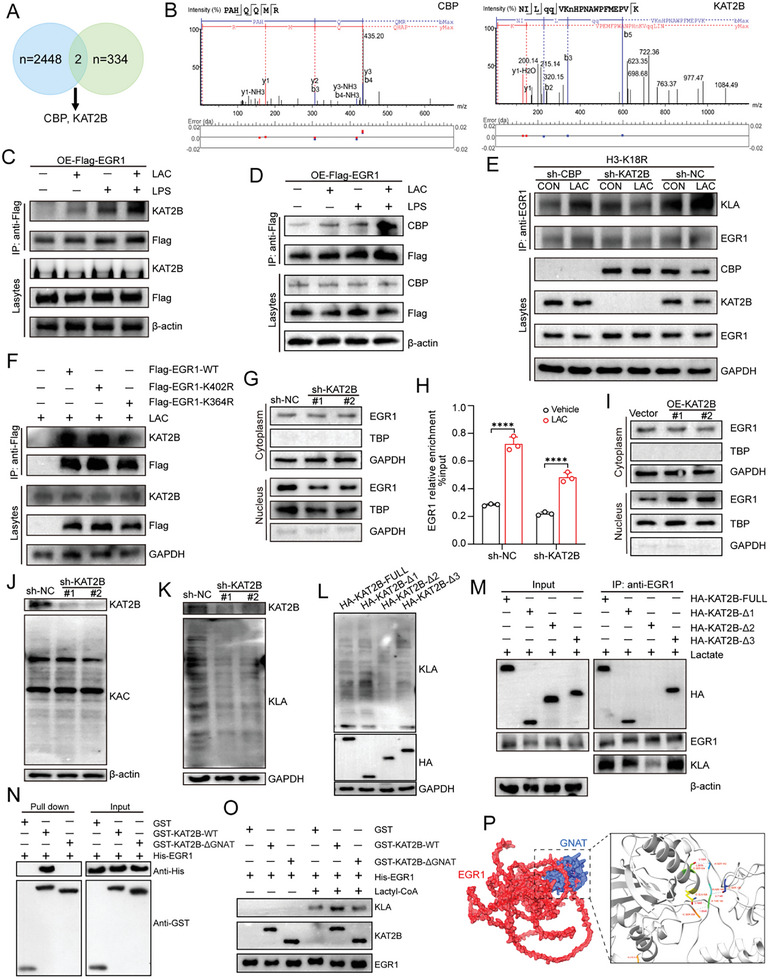
Identification of KAT2B as an EGR1 lactyltransferase. A) Identifying EGR1‐interacting acetyltransferase using IP in combination with LC‐MS/MS. B) The spectrograms showed mass spectroscopy‐identified CBP and KAT2B peptides. C) After MPMVECs be treated with LPS (12 ug mL^−1^ for 12 h), lactate (8 mM for 7 h), or both, co‐IP was performed to examine the interaction between Flag‐EGR1 protein and KAT2B in MPMVECs overexpressing the Flag‐EGR1. D) After MPMVECs be treated with LPS (12 ug mL^−1^ for 12 h), lactate (8 mM for 7 h), or both, co‐IP was performed to examine the interaction between Flag‐EGR1 protein and CBP in MPMVECs overexpressing the Flag‐EGR1. E) Kla levels of EGR1 in the H3‐K18R mutant MPMVECs transfected with scramble, CBP, or KAT2B‐targted shRNA. F) co‐IP was performed to examine the interaction between Flag‐EGR1 protein and KAT2B in MPMVECs overexpressed with WT, K402 or K364 point mutatio‐EGR1 after lactate stimulation (8 mM). G) After treatment with lactate (8 mM) for 7 h, cytoplasmic and nuclear EGR1 expression was measured in MPMVEC transfected with scramble or KAT2B‐targted shRNA. H) After transfected with scramble or KAT2B‐targted shRNA, ChIP‐qPCR detected EGR1 occupancy rates in the promoter region of HPSE after lactate stimulation (8 mM) (n = 3). I) After treatment with lactate (8 mM) for 7 h, cytoplasmic and nuclear EGR1 expression was measured in MPMVEC overexpressed with exogenous KAT2B. J) Global Kac levels of MPMVECs transfected with scramble or KAT2B‐targted shRNAs in the presence of lactate (8 mM). K) Global Kla levels of MPMVECs transfected with scramble or KAT2B‐targted shRNAs in the presence of lactate (8 mM). L) Global Kla levels of control vector or HA‐tagged KAT2B truncations‐overexpressed MPMVECs after lactate stimulation (8 mM). M) After treatment with lactate (8 mM for 7 h), co‐IP was performed to examine the lactylation levels of EGR1 and its interaction with HA‐KAT2B protein in control vector or HA‐tagged KAT2B truncations‐overexpressed MPMVECs. N) GST‐pull down assay was performed to examine the direct interaction between GST‐KAT2B‐WT, GST‐KAT2B‐ΔGNAT with His‐EGR1, respectively. O) In vitro EGR1 lactylation assay. Purified GST‐KAT2B‐WT or GST‐KAT2B‐ΔGNAT was incubated with purified His‐EGR1 with or without lactyl‐CoA. Kla levels of EGR1 were analyzed by WB. P) Molecular docking model of EGR1 (red) (PDB entry P08046) interacting with the GNAT‐domain of KAT2B (PDB entry Q9JHD1) (blue). All data were represented as the means±SD, *p < 0.05, **p < 0.01, ***p < 0.001, and ****p < 0.0001; ns, not significant.

To identify the enzymes that catalyze EGR1 lactylation, we performed coimmunoprecipitation (co‐IP) using an anti‐Flag antibody in FLAG‐tagged EGR1‐overexpressing MPMVECs and then performed LC‐MS/MS (**Figure** **7**A). A total of two acetyltransferases (CBP and KAT2B) were identified among the EGR1‐binding proteins (Figure 7B). The protein‐protein interaction network constructed with GeneMANIA confirmed the relationships among CBP, KAT2B and EGR1 (Figure S7A, Supporting Information). Subsequently, co‐IP assays further suggested that lactate and LPS significantly increased the binding of EGR1 to CBP and KAT2B (Figure 7C,D). We then over‐expressed or knockdown CBP and KAT2B separately to explore their potential influence on EGR1 lactylation. The IP results revealed that the Kla level of EGR1 was significantly increased only in the KAT2B‐overexpressing group (Figure S7B,C, Supporting Information), while KAT2B knockdown markedly reduced the lactylation of EGR1 (Figure 7E). The colocalization of EGR1 and KAT2B in MPMVECs further confirmed their interaction (Figure S7D, Supporting Information). However, the association between EGR1 and KAT2B decreased after EGR1‐K364R mutation (Figure 7F). These results demonstrated that KAT2B, not CBP, plays an important role in the lactylation of EGR1. Subsequently, we examined the impacts of KAT2B knockdown and overexpression on the nuclear translocation of EGR1 in MPMVECs (Figure S7E, Supporting Information). KAT2B knockdown significantly decreased the nuclear translocation of EGR1 and its enrichment in the HPSE promoter region following lactate treatment, while KAT2B overexpression had the opposite effects (Figure 7G‐I). In addition, the nuclear translocation of EGR1 induced by KAT2B overexpression was decreased in the presence of the EGR1‐K364R mutation (Figure S7F, Supporting Information). These findings suggest that KAT2B can regulate EGR1 nuclear translocation by mediating EGR1 lactylation.

The role of KAT2B, a member of the GNAT family of acetyltransferases, in regulating global acetylation and lactylation is unknown. We thus depleted KAT2B in MPMVECs and, surprisingly, the global acetylation level was not altered under the same lactate stimulation conditions (Figure 7J); in contrast, a substantial reduction in the global lactylation level was observed (Figure 7K). Furthermore, treatment with garcinol, an inhibitor of KAT2B,^[^ ^40^ ^]^ also effectively decreased the global lactylation level in MPMVECs (Figure S7G, Supporting Information). To map the region responsible for the activity of KAT2B as a Kla writer, we generated HA‐tagged full‐length KAT2B and three different KAT2B truncations, which lacked the PCAF_N domain (Δ56 to 308 aa), GNAT domain (Δ484 to 632 aa), or BROMO domain (Δ721 to 791 aa), based on the structural domain sequences of KAT2B (Figure S7H, Supporting Information). Both the PCAF and BROMO domains of KAT2B were dispensable for global lactylation, whereas the GNAT domain was necessary (Figure 7L). Furthermore, the co‐IP results showed that EGR1 bound selectively to the GNAT domain of KAT2B and that deleting the GNAT domain markedly decreased the EGR1 lactylation level compared with that observed for full‐length KAT2B (Figure 7M). As expected, the absence of the GNAT domain partially diminished the promoting effect of KAT2B on the nuclear translocation of EGR1 (Figure S7I, Supporting Information).

To investigate whether KAT2B‐GNAT domain directly bind with EGR1, we carried out an in vitro GST‐pull down assays by using purified GST‐KAT2B/KAT2B‐ΔGNAT and His‐EGR1. A strong binding was observed between His‐EGR1 with GST‐KAT2B but not GST‐KAT2B‐ΔGNAT, indicating that GNAT domain of KAT2B is the main site of connection with EGR1 (Figure 7N). To further investigate whether GNAT domain directly transfers lactyl‐CoA to EGR1, we carried out an in vitro lactylation assay by incubating purified GST‐KAT2B/KAT2B‐ΔGNAT and His‐EGR1 in the presence of lactyl‐CoA, a strong lactylation of His‐EGR1 was observed in the mixture of purified lactyl‐CoA and GST‐KAT8‐WT but not GST‐KAT2B‐ΔGNAT (Figure 7O), indicating that GNAT domain of KAT2B directly mediates EGR1 lactylation. Through the molecular docking model, we further identified the specific binding sites and modes between EGR1 and the GNAT domain of KAT2B (Figure 7P). In summary, our results indicate that the GNAT domain of KAT2B directly interacts with EGR1 and promotes its lactylation and nuclear localization.

### 2.9 EGR1 Knockout Attenuates Glycocalyx Degradation and ALI in Mice with Polymicrobial Sepsis

**Figure 8:**
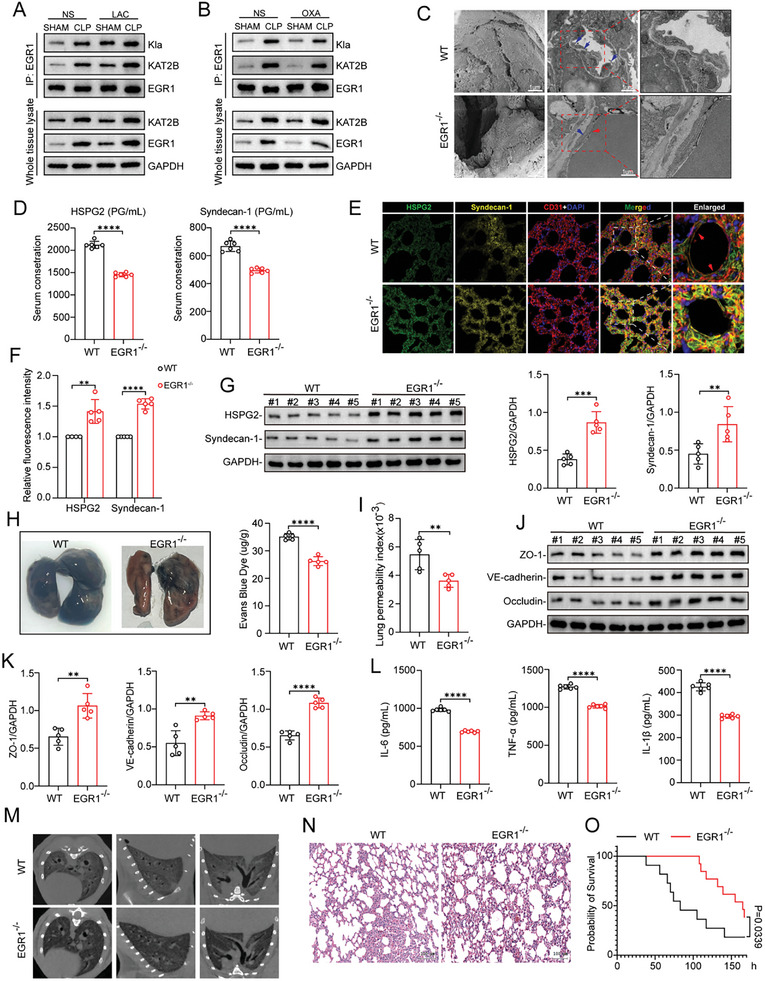
EGR1 knockout attenuates glycocalyx degradation and ALI in mice with polymicrobial sepsis. A) Expression of KAT2B and EGR1 in whole lung tissue lysate of mice injected with lactate (0.5 g kg^−1^ body weight) or not after Sham and CLP surgery. co‐IP was further performed to examine the lactylation levels of EGR1 and its interaction with KAT2B in tissue lysate. B) Expression of KAT2B and EGR1 in whole lung tissue lysate of mice injected with sodium oxamate (0.75 g kg^−1^ body weight) or not after Sham and CLP surgery. co‐IP was further performed to examine the lactylation levels of EGR1 and its interaction with KAT2B in tissue lysate. C) SEM and TEM imaging of the glycocalyx structure on the surface of pulmonary vascular using tracer lanthanum staining of in EGR1‐WT or EGR1^−/−^ mice after CLP surgery. Scale bar, 1µm. D) Serum concentration of HSPG2 and syndecan‐1 of EGR1‐WT or EGR1^−/−^ mice after CLP surgery (n = 6). E) Representative immunofluorescent staining images of HSPG2 (green) and syndecan‐1 (yellow) in endothelial surface of the lung tissues in EGR1‐WT or EGR1^−/−^ mice after CLP surgery. Endothelial surface were stained with CD31 (red), and nuclei were stained with DAPI (blue). Scale bar, 20 µm; F) quantification of fluorescence intensity was analyzed by ImageJ (n = 5 per group). G) Content of HSPG2 and syndecan‐1 in lung tissue of EGR1‐WT or EGR1^−/−^ mice after CLP surgery were detected by WB. The Right panel represents WB quantification (n = 5). H) Evans blue tracer in whole lung of EGR1‐WT or EGR1^−/−^ mice after CLP surgery. The Right panel represents Evans blue dye contents quantification (n = 5). I) Lung permeability index of EGR1‐WT or EGR1^−/−^ mice after CLP surgery (n = 5). J) Expression of ZO‐1 and Occludin in lung tissue of EGR1‐WT or EGR1^−/−^ mice after CLP surgery were detected by WB. K) WB quantification (n = 5). L) Concentration of IL‐6, TNF‐α, and IL‐1β in BALF of EGR1‐WT or EGR1^−/−^ mice after CLP surgery (n = 6). M) Detailed images of pulmonary inflammation of EGR1‐WT or EGR1^−/−^ mice after CLP surgery were derived with Micro‐CT. N) Representative HE staining of the lung tissue section of EGR1‐WT or EGR1^−/−^ mice after CLP surgery. Scale bar, 100 µm. O) Survival rates among EGR1‐WT or EGR1^−/−^ mice were compared by Kaplan‐Meier test. All data were represented as the means±SD, *p < 0.05, **p < 0.01, ***p < 0.001, and ****p < 0.0001; ns, not significant.

To elucidate whether EGR1 plays a role in degradation of the glycocalyx on the surface of the pulmonary vascular endothelium in vivo, we first evaluated the expression and lactylation of EGR1 in lung tissue after CLP. Compared with the Sham operation, CLP significantly promoted EGR1 expression and lactylation. Furthermore, lactate supplementation increased the expression of EGR1 and its interaction with KAT2B (**Figure** **8**A). Conversely, pretreatment with OXA decreased EGR1 expression and the interaction between EGR1 and KAT2B induced by CLP, leading to decreased EGR1 lactylation (Figure 8B). Next, we investigated whether EGR1 silencing can attenuate sepsis‐induced glycocalyx degradation and ALI in vivo. To this end, polymicrobial sepsis was induced by CLP surgery in EGR1 knockout (EGR1^−/−^) and WT mice, and lung tissue and blood samples were collected 24 h post‐surgery. The results of genotyping are shown in Figure S8A,B, Supporting Information. The expression of EGR1 in lung tissue was also examined by WB analysis (Figure S8C, Supporting Information). EGR1 knockout did not affect the level of lactate in lung tissue or serum (Figure S8D, Supporting Information). Glycocalyx degradation was less severe and the endothelial gaps were smaller in the pulmonary microvasculature in EGR1^−/−^ mice compared with WT mice (Figure 8C). Consistent with this finding, more sheddings of glycocalyx have been detected in the serum of WT mice (Figure 8D). Furthermore, the results of both IF staining and WB analysis showed that EGR1 silencing alleviated the sepsis‐induced decreases in HSPG2 and syndecan‐1 levels in lung tissue (Figure 8E‐G).

We then examined pulmonary vascular permeability in WT and EGR1^−/−^ mice following CLP surgery. EGR1 silencing significantly attenuated sepsis‐induced pulmonary vascular permeability (Figure 8H,I). We further measured the levels of endothelial junctional proteins, including ZO‐1, VE‐cadherin, and occludin, and found that EGR1 knockout markedly alleviated the decreases in their levels post‐CLP (Figure 8J,K). Moreover, EGR1 deficiency attenuated the pulmonary inflammatory response and degree of injury in septic mice, as evidenced by the results of proinflammatory factor quantification in BALF, imaging, and pathological analysis (Figure 8L‐N). Survival analysis revealed that compared with that of WT septic mice, the 7‐day survival rate of EGR1^−/−^ septic mice was increased by 59.3% (Figure 8O). Collectively, our results reveal that EGR1 plays an important role in S‐ALI by accelerating glycocalyx degradation on the surface of the pulmonary vascular endothelium.

## 3 Discussion

**Figure 9:**
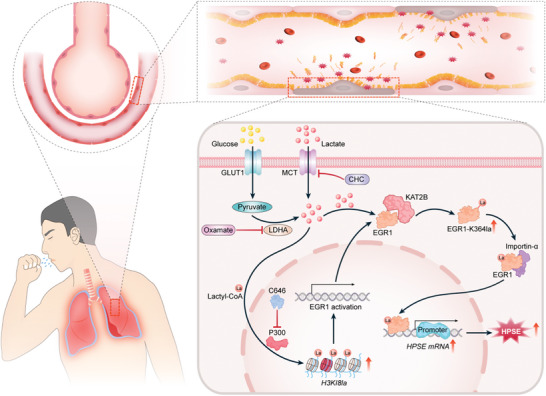
Schematic of enhanced glycolysis‐induced lactate overproduction promoting pulmonary endothelial glycocalyx degradation and ALI via histone H3K18 and EGR1 lactylation.

Despite major progress in diagnosis and treatment, S‐ARDS remains the most frequent complication arising during critical illness and can contribute to a poor clinical prognosis. Previous studies have suggested that epigenetic modifications are potential links connecting metabolic reprogramming and organ dysfunction in the context of sepsis. However, the mechanisms of this tandem process are incompletely understood. Our research suggests that during sepsis, excessive aerobic glucose metabolism leads to the generation of lactate, which accelerates the degradation of the glycocalyx on the surface of pulmonary microvessels, thereby resulting in increased vascular permeability and the exacerbation of lung injury. Specifically, an increase in H3K18la during S‐ALI can promote the transcription of EGR1 in MPMVECs, leading to high expression of HPSE. Simultaneously, KAT2B can directly mediate lactylation of EGR1 at K364, thereby facilitating the interaction between EGR1 and importin‐α as well as the nuclear localization of EGR1. Suppression of lactate production or EGR1 silencing attenuates glycocalyx degradation and ALI and improves the survival outcome of septic mice. A schematic model summarizing these mechanisms is presented in **Figure** **9**.

Accumulating evidence suggests that metabolic reprogramming plays a crucial role in the pathological damage caused by sepsis, including immune dysregulation and dysfunction of major organs.^[^ ^27^, ^41^ ^]^ Lactate, a primary product of glycolysis, is widely used clinically as a biomarker for the tissue perfusion status in septic patients.^[^ ^24^, ^25^ ^]^ Unlike its activity in other organs, glycolysis exhibits abnormal activity in lung tissue during sepsis.^[^ ^16^ ^]^ Li et al. recently reported that lactate can exacerbate sepsis‐induced hypervascular permeability by disrupting intercellular tight junctions through the endothelial surface receptor GRP81.^[^ ^17^, ^18^ ^]^ These results are similar to ours and suggest that lactate may be involved in the process of S‐ALI. Additionally, we isolated MPMVECs and investigated the potential role of lactate in the degradation of the extracellular glycocalyx by in vivo experiments and analysis of clinical samples. Unlike intercellular tight junctions, the vascular surface glycocalyx is very fragile. We found that treating MPMVECs with 8 mM lactate for 5 h sufficient to cause a decrease in the cell surface glycocalyx content. Although real‐time in vivo monitoring data are currently lacking, Wiesinger et al., using an atomic force microscopy nanoindentation technique, found that treating ex vivo rat aorta samples with heparinase I for 15 min resulted in a noticeable reduction in the endothelial glycocalyx thickness.^[^ ^42^ ^]^ Additionally, research by Nieuwdorp et al. demonstrated that low‐dose LPS stimulation for 24 h was sufficient to induce shedding of the vascular surface glycocalyx in healthy individuals.^[^ ^43^ ^]^ In contrast, the self‐repair of glycocalyx damage requires 5–7 days.^[^ ^44^ ^]^ Shedding of the glycocalyx exposes the unprotected endothelial surface and elicits the recruitment and activation of inflammatory cells.^[^ ^7^, ^11^ ^]^ Furthermore, the released glycocalyx fragments can act as highly potent damage‐associated molecular patterns (DAMPs), inducing endothelial damage.^[^ ^5^ ^]^ Together, these observations explain the lactate‐induced lung injury observed in the early stages of sepsis.

HPSE, an endogenous endo‐β‐D‐glucuronidase, is expressed ubiquitously in mammalian cells.^[^ ^45^ ^]^ It specifically recognizes and catalyzes the hydrolysis of β‐linkages between glucuronic acid and N‐acetylglucosamine residues in HSPGs.^[^ ^46^ ^]^ HPSE is produced predominantly by endothelial cells, and its production is stimulated by several factors, such as cytokines, growth factors, and metabolites.^[^ ^47^ ^]^ Upregulation of HPSE expression and activation is a common feature in the progression of several diseases, such as cancers, inflammatory diseases, and diabetes.^[^ ^47^ ^]^ In addition to bacterial and viral infections,^[^ ^48^ ^]^ the production of large amounts of inflammatory cytokines and DAMPs can also promote HPSE transcription via classical pathways such as the MEK/ERK and PI3K/AKT pathways,^[^ ^49^ ^]^ in turn exacerbating glycocalyx degradation and endothelial barrier dysfunction. Consistent with previous studies, we observed significant increases in the level and activity of HPSE in the blood of patients with S‐ARDS, which correlated with disease severity.^[^ ^19^, ^50^ ^]^ This study provides the first report that lactate can act as a novel regulatory factor that promotes HPSE expression through PTMs. However, RNA‐seq analysis indicated that lactate is extensively involved in the signaling regulatory network of endothelial cells. Thus, we cannot exclude the possibility that other signaling pathways may also participate in HPSE regulation. It has been reported that HPSE performs both enzymatic and nonenzymatic functions in a pH‐dependent manner. HPSE showed favorable endoglycosidase activity for optimal binding of HSPGs at pH values ranging from 5.0 to 6.0,^[^ ^51^ ^]^ which partially explains its increased enzyme activity at sites of lactate accumulation. However, we observed that the same concentration of NaLa similarly induced the upregulation of HPSE activity in MPMVECs in vitro, indicating that the change in pH cannot fully explain the increase in HPSE activity. Although how lactate regulates the activity of HPSE remains unknown, based on previous research, it can be predicted that lactate may mediate the modification of the spatial conformation of HPSE, which ultimately alters its interaction with substrate proteins.^[^ ^52^ ^]^

EGR1, an important TF of HPSE, contributes to inflammation‐associated lung diseases.^[^ ^53^ ^]^ It has been reported that EGR1 activation relies predominantly on the MAPK and cAMP signaling pathways^[^ ^54^ ^]^ and is involved in apoptosis, autophagy, and fibrosis.^[^ ^55^, ^56^, ^57^ ^]^ In addition, a derived engineered extracellular vesicle developed by Gu et al. that targets the EGR1 gene was found to significantly attenuate pulmonary endothelial barrier damage in mice with ALI.^[^ ^58^ ^]^ The present study revealed that lactate administration significantly increased EGR1 expression in lung tissue and MPMVECs. In vivo, silencing EGR1 alleviated sepsis‐induced degradation of the vascular endothelial glycocalyx and lung injury. Recent studies have shown that lactate can participate in the epigenetic regulation of gene transcription via histone modifications^[^ ^20^ ^]^ and plays a crucial role in sepsis‐associated immune dysregulation and acute kidney injury.^[^ ^21^, ^23^ ^]^ In the present study, we observed an increased global lactylation level in lung tissue during sepsis. Subsequent CUT&Tag assays proved that H3K18la directly binds to the EGR1 promoter region. Both interference with lactate production and inhibition of P300 reduced the H3K18la and HPSE levels. Diverse PTMs, including SUMOylation, ubiquitination, and acetylation,^[^ ^59^, ^60^, ^61^ ^]^ also influence the activity and function of EGR1 itself. By LC‒MS/MS, we confirmed multiple lactylation sites in EGR1 and proved that the direct lactylation of EGR1 promoted its nuclear translocation. This finding is similar to the results of Fan et al., who reported that CBP/P300‐mediated lactylation and nuclear localization of Snail1 accelerated endothelial‐to‐mesenchymal transition after myocardial infarction.^[^ ^36^ ^]^ It has been reported that mutations at basic residues of multiple adjacent C2H2 ZFDs often change the conformation and function of target proteins, for example, by mediating their interactions with other molecules and their subcellular localization.^[^ ^38^, ^62^ ^]^ Analysis of the crystal structure of EGR1 showed that EGR1 consists of three highly conserved DNA structural domains encoding C2H2 ZFDs.^[^ ^53^ ^]^ In the present study, a significant increase in the interaction between K364la‐modified EGR1 and importin‐α induced by lactate treatment was demonstrated in vitro, and the EGR1 K364R mutation suppressed this binding and attenuated EGR1 nuclear translocation. Coincidentally, a recent study also confirmed that lactylation of XRCC1 leads to changes in its surface charge, thereby promoting its binding to importin‐α and ultimately resulting in the nuclear translocation of XRCC1.^[^ ^63^ ^]^ Together, our findings suggest that H3K18la‐induced EGR1 expression and EGR1 lactylation are important mechanisms through which lactate promotes glycocalyx degradation via the activation of HPSE during sepsis. Unfortunately, in this study, we did not further investigate whether lactylation of EGR1 exhibits disease specificity. Future studies could explore the differences in modification sites, distribution, and functions of EGR1 under different disease states.

Although the lactylation of numerous nonhistone proteins, such as Ezrin, Snail1, HMGB1, and Fis1, has been reported,^[^ ^21^, ^23^, ^36^, ^64^ ^]^ knowledge of these modification processes is insufficient. Unlike histone lactylation, the key enzymes mediating the lactylation of non‐histone proteins remain largely unclear. KAT2B, a member of the GNAT protein family, plays various roles in the development of both tumors and inflammatory diseases, such as sepsis,^[^ ^65^, ^66^ ^]^ through acetylation. In this study, we showed that KAT8 could also act as a lactyltransferase and mediate EGR1 lactylation. These findings help elucidate the regulatory mechanism of KAT2B function in sepsis, indicating that the lactylation‐mediated progression of S‐ALI involves a complex and diverse regulatory network. In addition, we noted that high lactic acid conditions amplified the functional roles of KAT2B‐lactylated proteins rather than acetylation, which indicated that KAT2B performs diverse functions that may be highly state‐ and context dependent. However, it should be noted that we did not fully clarify the lactylation regulatory mechanism mediated by KAT2B and its downstream protein targets. Thus, other KAT2B‐lactylated proteins may also participate in S‐ALI, as evidenced by the increase in the global lactylation level induced by KAT2B, and these proteins deserve further exploration in specific contexts.

In summary, our study revealed a previously unrecognized role of glycolysis‐derived lactate in promoting glycocalyx degradation and ALI during sepsis. On the one hand, lactate promoted an increase in H3K18la and activated EGR1 expression; on the other hand, it induced the lactylation and nuclear translocation of EGR1, thereby accelerating glycocalyx degradation by upregulating HPSE expression. These findings illuminate a novel mechanism responsible for glycocalyx degradation and extend our understanding of the important biological function of lactate in S‐ALI.

## 4 Experimental Section

### Patients and Clinical Blood Samples

Whole blood samples from the S‐ARDS patients were collected from December 2022 to November 2023 by the Department of Respiratory and Critical Care Medicine, Anhui Chest Hospital, within the first 12 h from admission. Sepsis and ARDS were diagnosed by Sepsis‐3.0 and Berlin Definition criteria, respectively.^[^ ^1^, ^67^ ^]^ Patients with the following characteristics were excluded: 1) Incomplete information; 2) Presence of malignant tumors or tuberculosis; 3) Unacceptable blood samples. Blood samples from healthy controls (HCs) was obtained from Medical Examination Centre of Anhui Chest Hospital. Eventually, a total of 24 HCs and 65 S‐ARDS patients were included. This study was performed in accordance with the tenets of the Declaration of Helsinki, and was approved by the Ethics Committee of the Anhui Chest Hospital (KJ2023‐40).

### Animals

8–12‐week‐old male mice were used in all experiments in this study. WT C57BL/6J mice were purchased from the Institute of Health and Medicine, Hefei Comprehensive National Science Center (Hefei, China). EGR1 knockout (EGR1^−/−^) mice were provided by Cyagen Biosciences Inc (Suzhou, China), and the gRNA target sequences were as follows:gRNA‐A1: TAATGCGCGCCCCCGACCCTGGG, gRNA‐A2: GGACAATTGAAATTTGCTAAAGG, gRNA‐B1: CAGGGATGTCCCGCCGCCCAGGG, gRNA‐B2: GACAATTGAAATTTGCTAAAGGG. All mice were maintained and bred at the animal research platform at Hefei Comprehensive National Science Center. Genotyping of mice were performed by polymerase chain reaction (PCR) using tail‐snip DNA. Age‐and sex‐matched male mice aged 8–12 weeks were used in experiments. And the experiments were conducted in a blinded manner. Animal experiments strictly complied with the Experimental Animal Management Ordinance of China, and were approved by the Institutional Animal Ethics Committee of the Hefei Comprehensive National Science Center (IHM‐AP‐2024‐004).

### CLP‐Induced Polymicrobial Sepsis Model

Mice were randomly divided into different treatment groups, and the experimenter was blinded. Polymicrobial sepsis was induced by CLP in mice as described previously.^[^ ^18^ ^]^ Briefly, following anesthesia with 4% isoflurane gas, the lower abdominal of mice was shaved and disinfected, a 1 cm skin incision was made along the midline of the abdomen and the cecum was exteriorized. The cecum was ligated between the third and fourth vascular arcade with a 4‐0 silk suture and punctured with a 21‐gauge needle, followed pushed the fecal material from punctured holes. Sham‐operated mice were used as controls. Following surgery, a single dose of resuscitative fluid was administrated by subcutaneous injection. Mice blood and lung tissue samples were collected at the indicated time points as pre‐established protocol and stored at −80 °C for further experiments.

### Variable Extraction from the MIMIC‐IV Database

The cohort of patients used in this present study was from MIMIC‐IV 2.0, published on 12 June 2022, in which comprehensive electronic medical record for over 40 000 patients admitted to intensive care units at the Beth Israel Deaconess Medical Center in Boston, Massachusetts, between 2008 and 2019 were contained. All patients with sepsis were initially included in this study based on Sepsis‐3.0,^[^ ^1^ ^]^ the SOFA score greater than 2 points based on confirmed or suspected infections. In contrast, the suspected infections have defined the records of empiric antibiotic therapy prior to or within 3 days after culture collection in the MIMIC‐IV database. While, an ARDS diagnosis was made according to the Berlin Definition criteria.^[^ ^67^ ^]^ If patients were admitted more than once, only their first stay was included in the analysis. Patients were excluded from the study if they were younger than 18 years, were discharged or died within 48 h after ICU admission, no lactate test record. Laboratory test data within the first 24 h after ICU admission, clinical characteristics and survival outcomes were extracted. MIMIC‐IV database was approved by the Institutional Review Board of the Massachusetts Institute of Technology. One author (Zongqing Lu) in this study obtained access and was responsible for the data extraction (certification no. 38 455 175).

### In Vivo Treatment

To investigate whether increased serum lactate levels could accelerate the glycocalyx degradation on the surface of pulmonary microvasculature, lactic acid (Sigma‐Aldrich) was injected intraperitoneally (pH 6.8, 0.5 g kg^−1^ body weight) 6 h following CLP/sham surgery. To suppress lactate production, sodium oxamate (Sigma‐Aldrich) (0.75 g kg^−1^ body weight) was intraperitoneally injected 3 h before CLP or sham surgical operation.

### Isolation and Identification of Primary MPMVECs

MPMVECs were isolated and cultured from mice according to the previous method of our project team.^[^ ^30^ ^]^ Briefly, following anesthesia with 4% isoflurane gas, blood was collected via heart puncture from male SPF C57BL/6 mice, then fresh lung tissues were isolated and immediately immersed in the ice‐cold Dulbecco's modified Eagle medium (DMEM) under sterile conditions. After removing the visceral pleura of the lung tissues, the thin strips of the surrounding lung lobe were obtained and carefully cut into 1–2 mm^2^ small pieces. Then, the chopped lung tissues were dispersed into a cellular culture flask and incubated with DMEM, supplemented with 20% FBS, 1% endothelial cell‐derived growth factor, and 1% P/S. After incubation for 60 h in 37 °C, 5% CO_2_ humidified incubator, the minced lung tissues were removed, and the MPMVECs continued to incubate in the incubator until cells reached 80–90% confluence. The purity of the MPMVECs was confirmed by flow cytometry with anti‐CD31.

### Culture of MPMVECs

Following isolation, MPMVECs were cultured using complete growth medium in 37 °C, 5% CO2 humidified incubator, and the medium was replaced every 2 days. To mimic the inflammatory environment in vitro, the MPMVECs were stimulated with 12 ug mL^−1^ *E. coli*‐LPS for 12 h, then stimulated with 8 mM lactate or PBS for 7 h. In separate experiments, MPMVECs were pre‐treated with OXA (dissolved in PBS, 20 mM) for 3 h, an MCT inhibitor (CHC, dissolved in PBS, 3 mM) for 1 h, an P300 inhibitor (C646, dissolved in PBS, 5uM) for 3 h, or an KAT2B inhibitor (Garcinol, dissolved in DMSO, 4–32 uM) for 1 h, followed by lactate (8 mM) stimulation for 7 h or LPS (12 ug mL^−1^) for 12 h.

### Micro‐Computed Tomography Analysis

To assess the degrees of ALI in vivo, mice were subjected to thorax irradiation and computed tomography (CT) monitoring by Hiscan XM Micro CT Imaging System (Hiscan Information Technology Co.,Ltd, China). The X‐Ray tube settings were 60 kV and 134 uA and images were acquired at 50um resolution. A 0.5° rotation step through a 360°angular range with 50 ms exposure per step was used. Hiscan Analyzer software version 3.0 (Hiscan Information Technology Co.,Ltd, China) was used for image processing and 3D reconstruction.

### H&E Staining and Evaluation of Lung Injury Severity

Lung tissues were fixed in 4% paraformaldehyde (PFA,Servicebio, G1101) and then embedded in paraffin. Formalin‐fixed and paraffin‐embedded tissues were cut into 4 mm thick sections. Lung tissue sections were stained with hematoxylin and eosin (H&E) to visualize the damage. The lung injury score was evaluated based on the following criteria: oedema (0–4), hemorrhage (0–4), neutrophil infiltration (0–4), and hyaline membrane formation (0–4). Five fields of view were randomly selected on each slide to count the damage score, and the mean was taken.

### BALF Cell Counting and Classification

Following anesthesia with 4% isoflurane gas, the lungs were infused with 0.5 mL pre‐cooled saline via a tube, and bronchoalveolar lavage was carried out for three times and collected into one tube. 50 uL alveolar lavage fluid was used for counting by XT‐2000i (Sysmex, Japan). Besides, cells in BALF were classified by stained modified Giemsa staining (Beyotime Biotechnology, China).

### In Vivo Vascular Permeability Assay

Evans blue dye (EBD) extravasation assay and pulmonary permeability index were used to evaluate the mice pulmonary vascular permeability. For EBD extravasation assay, EBD (50 mg kg^−1^) in 200 uL of 0.9% saline was injected into the mouse tail vein 1 h before sacrifice. To wash intravascular EBD, this work performed transcardiac perfusion with 0.9% saline solution and obtained the lungs. Then the tissue was weighed, homogenized, and incubated with 200 uL of formamide (56 °C for 24 h) to extracted EBD. Finally, the supernatants were collected, and the concentration of Evans blue was estimated by spectrophotometer at 620 nm. For pulmonary permeability index calculation, this work first tested the protein content of plasma and BALF by BCA protein assay kit (Beyotime Biotechnology, P0012). Pulmonary permeability index calculation was calculated as the BALF protein content divided by the plasma protein content.

### Electron Microscopy

TEM and SEM analyses of the endothelial glycocalyx were performed as previously described.^[^ ^68^, ^69^ ^]^ Briefly, mice were anesthetized and perfused with a solution composed of 2% glutaraldehyde, 2% sucrose, 0.1 M sodium cacodylate buffer (pH 7.3), and 2% lanthanum nitrate through a cannula placed in the left ventricle 24 h following CLP/Sham surgery. Prior to perfusion, a surgical incision was made in the right atrial appendage and the neck was ligated with a silk suture. And, a perfusion pump was utilized to administer the injection at a constant rate of 1 mL per minute. The lung was harvested and gently diced (1mm^3^); immediately immersed in a phosphate‐buffered fixative (pH 7.3) composed of 2% glutaraldehyde, 0.05% Alcian blue 8GX, and 30 mmol L^−1^ MgCl_2_ for 2 h, and then soaked in phosphate‐buffered fixative with 2% lanthanum nitrate, 2% glutaraldehyde, and 2% sucrose overnight (4 °C). For SEM analysis, tissues were washed in alkaline (0.03 mol L^−1^ NaOH) 2% sucrose solution. The samples were then dehydrated through a graded ethanol series. After ion sputtering (Cressington‐1080auto, UK), the specimens were examined using SEM (Zeiss‐GeminiSEM 300, Germany). In addition, to further elemental analysis of each sample, energy‐dispersive X‐ray spectroscopy was performed under SEM. For TEM analysis, the specimens were postfixed with 1% aqueous osmium tetroxide and 1% lanthanum nitrate for 1 h, dehydrated through a graded ethanol series, and embedded in Epon812. Ultrathin sections stained with uranyl acetate 15 min and lead citrate 2 min were then examined using TEM (Thermo Scientific‐Talos L120C G2, USA).

### In Vitro Permeability Assay

The Endothelial barrier permeability was assessed by trans‐endothelial electrical resistance (TEER) which calculated by an electric cell‐substrate impedance sensing (ECIS) system (Millipore Millicell ERS‐2, USA). MPMVECs were seeded in the upper chamber of Transwell inserts (Corning, USA) and cultured into confluent monolayers using complete growth medium. Baseline TEER was measured 1 h before experiments. Then this work measured the TEER 12 h after LPS treatment and 7 h after Lac/Nala treatment to evaluate the changes of MPMVECs permeability.

### Enzyme‐Linked Immunosorbent Assay

Following the instructions of manufactures, the HSPG2 and syndecan‐1 levels in S‐ARDS patients and HCs were measure by SDC1/Syndecan‐1 ELISA kit (Sigma‐Aldrich, RAB0736) and Heparan Sulfate Proteoglycan 2 ELISA kit (Cloud‐Clone Crop, SEC748Hu). Mice serum, lung tissues, and cells lactate levels were measure by L‐Lactic Acid (LA) Colorimetric Assay Kit (Elabscience, E‐BC‐K044‐S). Mice serum concentration of HSPG2 and syndecan‐1 were measured by Heparan Sulfate Proteoglycan 2 ELISA kit (Cloud‐Clone Crop, SEC748Mu) and Syndecan‐1(SDC1) ELISA kit (CUSABIO, CSB‐EL020888MO). The ATP levels in mice lung tissue was quantified by ATP assay kit (Nanjing Jiancheng Bioengineering Institute, A095‐1‐1). The concentration of IL‐1β, IL‐6 and TNF‐α in mice BALF were measured by Mouse IL‐1β (Interleukin 1 Beta) ELISA Kit (Elabscience, E‐EL‐M0037), Mouse IL‐6 (Interleukin 6) ELISA Kit (Elabscience, E‐EL‐M0044) and CLIA Kit for Tumor Necrosis Factor Alpha (TNFa) (Cloud‐Clone Crop, SCA133Mu), respectively.

### Heparanase Enzymatic Content and Activity Assay

The Heparanase ELISA kit was utilized for quantifying the HPSE content in clinical serum samples (Cloud‐Clone Crop, SEA711Hu). HPSE activity was measured using a Heparan Degrading Enzyme Assay Kit (Takara, MK412), which employs the Domain Oriented Capture method involving the cell binding domain‐basic fibroblast growth factor (CBD‐bFGF) complex. In this method, CBD‐bFGF binds to a microtiter plate through interaction with an anti‐fibronectin antibody recognizing the CBD region. Furthermore, biotinylated heparan sulfate serves as a substrate for the enzyme. Upon degradation by the heparan sulfate degrading enzyme, the ability of heparan sulfate to bind to bFGF is diminished. The enzymatic activity can be assessed through quantitative analysis of the binding of undegraded heparan sulfate to bFGF in the presence and absence of the sample.

### Immunofluorescence Staining

Mice lung tissue samples were fixed in 4% PFA, embedded in paraffin and then cut at a 4‐mm thickness. Paraffin sections were deparaffinized and boiled in the Tris‐EDTA buffer (pH 8.0, Servicebio, G1206) for 2.5 min in a pressure cooker. For immunofluorescence staining of cells, MPMVECs were washed with PBS, fixed in 4% PFA, permeabilized with 0.1% Triton X‐100 (Beyotime Biotechnology, P0096), and blocked with 3% bovine serum albumin (dissolved in PBS, Beyotime Biotechnology, ST2249) for 30 min at 37 °C. Cells were then incubated with primary antibodies diluted in primary antibody dilution buffer for immunol staining (Beyotime Biotechnology, P0262) overnight at 4 °C. Cells was slow rewarmed 1 h at room temperature in the following day and washed with PBS, then incubated for 1 h with fluorescent secondary (1:200) antibody. Slides were mounted using antifade mounting medium containing DAPI (Biosharp, BL739) for nuclear staining. Images were acquired using LSM‐880 laser scanning confocal microscope (Zeiss, Germany) and analyzed by ImageJ and ZEN 2.0 imaging software (blue version, Zeiss).

### Cell Viability Assay

Cell viability was assessed using a Cell Counting Kit‐8 (CCK‐8) assay kit (APE×BIO, K1018). MPMVECs were cultured in 96‐well plates and exposed to varying concentrations and time of lactate. Subsequently, 10 µL of CCK‐8 reagent was added to each well and incubated at 37 °C for 2 h in the absence of light. The optical density of each well was then determined at a wavelength of 450 nm using a microplate reader (PerkinElmer, USA).

### Apoptosis Assay

MPMVECs treated with varying concentrations and time of lactate were stained with annexin V and propidium iodide using the FITC Annexin V Apoptosis Detection Kit (Biosharp, BL107B) and analyzed by CytoFLEX SRT‐flow cytometer (Beckman Coulter, USA).

### Nuclear‐Cytoplasmic Protein Separation

MPMVECs were washed with PBS, scraped off plates using a cell scraper and spun down at 300 x g for 10 min at 4 °C. The nuclear‐cytoplasmic protein assay was performed using the Nuclear and Cytoplasmic Protein Extraction Kit (Beyotime Biotechnology, P0027). Extracted nuclear and cytoplasmic proteins were immunoblotted with the indicated antibodies.

### Immunoprecipitation

Immunoprecipitation was conducted following the protocol provided by the manufacturer for the PierceTM Classic Magnetic IP/Co‐IP Kit (Thermo Fisher, 88 804). The primary antibody was initially incubated with cell lysate at 4 °C overnight to facilitate the formation of the immune complex. Subsequently, Pierce Protein A/G Magnetic Beads were mixed with the antigen sample and antibody mixture at room temperature for 1 h. The elution of the bound protein was then carried out by 1 x loading buffer for subsequent western blot analysis or mass spectrometry (MS).

### Silver Staining and Mass Spectrometry

Silver staining of SDS‐PAGE was performed with the Fast Silver Stain Kit (Beyotime Biotechnology, P0017S) following the manufacturer's recommendations. It involves fixing the gel to immobilize proteins, treating it with silver ions to bind proteins, and then developing with a reducing agent to reveal protein bands. Finally, a stop solution is applied to halt the reaction and prevent over‐staining. Band strips were then cut according to molecular weights (black boxed area). Proteins were identified by MS performed by the Personal Biotechnology Co.,Ltd (Shanghai, China). A total of 15 histone H3‐related peptides were identified.

### Liquid Chromatography‐Tandem Mass Spectrometry

LC‐MS/MS was employed to determine the lactylation sites of EGR1 and its interacting proteins in MPMVECs. For lactylation sites of EGR1 identification, cell samples were homogenized by a high intensity ultrasonic processor (Scientz) in lysis buffer (8 M urea, 3 uM TSA, 50 mM NAM, 2 mM EDTA, and 1% protease inhibitor cocktail) on ice. Then the lysed material was centrifuged at 12 000 g for 10 min at 4 °C. The supernatant was collected in new tubes, and the protein concentration was determined by BCA assay. In order to facilitate the reduction of disulfide bonds, the extracted proteins were treated with 5 mM dithiothreitol at 37 °C for 60 min, followed by alkylation with 10 mM iodoacetamide for 45 min at room temperature in the absence of light for digestion. Subsequently, the urea concentration in the protein solution was reduced to below 2 M through the addition of ammonium bicarbonate (25 mM). The protein underwent digestion by trypsin at a trypsin/protein mass ratio of 1/50 at 37 °C overnight, followed adjust pH less than 3 by adding formic acid. Finally, tryptic peptides were desalted by ultrafiltration (Millipore, 10KD). To enrich the lactylated peptides, tryptic peptides dissolved in NETN buffer (100 mM NaCl, 1 mM EDTA, 50 mM Tris‐HCl, 0.5% NP‐40, pH = 8.0), were incubated with agarose beads coupled to Anti‐L‐Lactyl Lysine antibody (PTM‐1401RM, PTM Bio) overnight at 4 °C with gentle shaking. Subsequently, the beads underwent three washes with NetN buffer followed by one wash with dd H_2_O. The lactylated peptides were eluted with 0.1% trifluoroacetic acid. Finally, the peptides were desalted by C18 ZipTips (Millipore, ZTC18 M). The liquid phase solution A was 0.1% formic acid acetonitrile water solution, while the solution B was 0.1% formic acid acetonitrile aqueous solution (84% acetonitrile). Peptides were loaded onto Zorbax 300SB‐C18 peptide traps (Agilent Technologies), followed separated by analytical column (0.15mm*150 mm, RP‐C18, Column Technology Inc.) according to a gradient from 4–50% solution B over 50 min, 50–100% in 4 min then holding at 80% for the last 6 min. Peptides were subjected to capillary source followed by the Q Exactive HF‐X (Thermo scientific) mass spectrometry.

For EGR1 interacting proteins determination, CO‐IP MS was used. Briefly, this work first overexpressed Flag‐EGR1 in MPMVECs, followed stimulation by lactate (8 mM) 7 h. Then PierceTM Classic Magnetic IP/Co‐IP Kit was used to extract the potential proteins which interacted with EGR1 by anti‐Flag antibody. The peptides extracted from eluted protein complex digestion, was were re‐dissolved in solvent A (A: 0.1% formic acid in water) and analyzed by Orbitrap Exploris 480 coupled to an EASY‐nanoLC 1200 system (Thermo Fisher Scientific). 1 uL peptide sample was loaded onto a 25 cm analytical column (75 um inner diameter, 1.9 um resin (Dr Maisch)) and separated with 60min‐gradient starting at 2.2% buffer B (80% ACN with 0.1% FA) followed by a stepwise increase to 50% in 51 min, 90% in 3.5 min and stayed there for 5.5 min. The column flow rate was maintained at 350 nL min^−1^ with the column temperature of 40 °C. The electrospray voltage was set to 2 kV.

### Protein Identification

MaxQuant search engine (1.5.5.1) was used to identify the lactylated sites in EGR1. Tandem mass spectra were searched against the uniprotkb_Mus_musculus (version 2023, 55 086 entries) database concatenated with reverse decoy database. Additionally, the peptides mass tolerance was set as 20 ppm in first search and 5 ppm in main search, and the mass tolerance for fragment ions was set as 0.1Da. Carbamidomethyl‐cysteine was specified as fixed modification, and lactylation were specified as variable modifications. FDR was adjusted to <1%.

PEAKS Studio (10.6) was used to identify the interaction protein with EGR1. The database was uniprotkb_Mus_musculus (version2022, 21 992 entries). Trypsin was set as the digestion enzyme and Semi‐specific was specified as the digest type. PEAKS DB were searched with a fragment ion mass tolerance of 0.02 Da and a parent ion tolerance of 10ppm. The max missed cleavages was 2. Carbamidomethyl on Cysteine was specified as the fixed modification. Oxidation on methionine, Deamidation on asparagine and glutamine, Acetylation on Protein N‐term were specified as the variable modifications. The peptides with 1% FDR and the proteins with 1% FDR and containing at least 1 unique peptide were filtered.

### CUT&Tag

CUT&Tag libraries amplification and purification were conducted according to the instructions of the NovoNGS CUT&Tag 4.0 High‐Sensitivity Kit (for Illumina) (NovoProtein, N259‐YH01). Briefly, counted 50 000 MPMVECs, treated by lactate, were immobilized on Concanavalin A magnetic beads and permeabilized. After being resuspended in an antibody buffer, the cells underwent sequential treatment with primary and secondary antibodies directed against H3K18la (PTM‐1427RM, PTM Bio) and H3K18ac (PTM‐114RM, PTM Bio). The samples and pAG‐Transposome were incubated simultaneously. DNA was isolated, amplified, and purified after transposon activation and tagmentation in order to construct the library. Using NovoNGS DNA Clean Beads, purification processes were completed following the construction of the sequencing library. The DNA libraries were sequenced on the Illumina PE150 platform to a depth of 20 G per sample. DNA Fragments are sequenced and analyzed by APExBIO Technology LLC (Shanghai, China). Visualization of called peaks was conducted using IGV v2.14.1.

### RNA‐Seq and Analysis

TRIzol was utilized in compliance with the manufacturer's instructions to extract total RNA from MPMVECs. After that, RNA was digested for reverse transcription, and rRNA was degraded. The DNBSEQ platform was used to create and sequence the cDNA libraries. DEGseq was used to identify differentially expressed genes, with q‐value < 0.05 and |log2Fc| >1. Sequencing and bioinformatics analysis were performed by Huada Gene Company (Wuhan, China).

### Ingenuity Pathway Analysis

IPA was used to identify the target of upstream regulator of HPSE in Ingenuity Knowledge Base (Ingenuity Systems, USA) based on RNA‐seq data. Subsequently, Causal Network Analytics and BioProfiler were used to visualized the gene regulatory networks and provide plausible explanations for the underlying latent relationship.

### Western Blot Analysis

The methodology for Western blot analysis involved denaturing cells and tissues followed by electrophoresis using SDS‐PAGE and transfer onto PVDF membranes (Millipore, USA). Protein concentration was quantified using the Pierce BCA protein assay kit (Beyotime Biotechnology, P0012S). Subsequently, the membranes were blocked with 5% skimmed milk for 1.5 h and then incubated with primary antibodies overnight at 4 °C. Secondary antibodies were applied at room temperature for 1 h before detection using chemiluminescence (Tanon5200, China). Analysis of the Western blot protein bands was conducted using Image J software. The antibodies used were shown in Table S4, Supporting Information.

### RNA Extraction and Real‐Time Quantitative PCR

Total RNA from tissues or cells were extracted using HiPure Total RNA Mini Kit (Magen, R4111), and reverse transcribed into complementary DNA (cDNA) using a HiScript IV RT SuperMix for qPCR (+gDNA wiper) Kit (Vazyme Biotech Co, R423). RT‐qPCR was performed with ChamQ SYBR qPCR Master Mix Kit (Vazyme Biotech Co, Q311) by Roche LightCycler 480 II (Roche, Swiss), and the amount of RNA was analyzed using Nanodrop one (2505) (ThermoFisher, USA). Genes expression levels were normalized to the signals of GAPDH expression. The primer sequences are listed in Table S5, Supporting Information.

### Molecular Docking

The crystal structures of GNAT‐domain of KAT2B and EGR1 of mice were accessed from the AlphaFold Protein Structure Database (https://alphafold.ebi.ac.uk/). The obtained protein crystals were processed using the H++ 4.0 server (http://newbiophysics.cs.vt.edu/H++/) for protonation, then dealt by UCSF Chimera software to assign the Amber14SB charge. The Discovery studio was used to dock the GNAT‐domain of SOCS1 with EGR1, all the sampled binding modes were evaluated by iterative knowledge‐based scoring function ITScorePP. A larger absolute value of the docking score indicated a stronger binding ability. Then, this work conducted credibility analysis by calculating the confidence score. The configuration with the best docking and confidence score was visualized by LigPlot 2.1 (EMBL‐EBI, UK).

### Chromatin Immunoprecipitation Assay

ChIP assays were conducted by using the BeyoChIPTM ChIP Assay Kit (Beyotime, P2080S). Briefly, about 1 × 10^6^ MPMVECs cross‐linked with formaldehyde 10 min at 37 °C, and chromatin fragmentation was carried out by ultrasonic breaking according to the protocol provided. The above‐prepared diluted soluble chromatin solution was then incubated with primary antibody overnight at 4 °C with rotation. Normal IgG was used to determine nonspecific bindings. The above mixtures were next incubated with Protein A/G Magnetic Beads for 60 min on a shaker at 4 °C, and the protein‐DNA complexes were eluted out after washes. The eluted DNA was subjected to ChIP‐PCR or ChIP‐qPCR with primers of indicated promoter region (Table S5, Supporting Information).

### Protein Expression and Purification

For His‐tagged EGR1 protein expression, E. coli BL21(DE3) transformed with the pET28a‐EGR1 plasmids was grown at 37 °C overnight and induced by 1 mM IPTG at 18 °C for 1 h. The cells were collected by centrifugation at 1000 g and fragmented by sonication in His‐lysis buffer (25 mM Tris‐HCl, pH 8.0, 300 mM NaCl, 20 mM imidazole, 5% Glycerol (v/v), 1 mM TCEP). The lysates were centrifuged, and the supernatant was collected, followed loaded onto a pre‐equilibrated HisPurTM Ni‐NTA resin (5 mL, Thermo Fisher, cat 90 099). Then, the column was washed by His‐lysis buffer (without Glycerol (v/v) and TCEP) and eluted by His‐elution buffer (25 mM Tris‐HCl, pH 8.0, 300 mM NaCl, 400 mM imidazole). Finally, purified proteins were buffer exchanged into PBS. For GST‐tagged KAT2B or KAT2B‐aa484‐632 protein expression, E. coli BL21(DE3) transformed with the pGEX‐4T‐1‐KAT2B/KAT2B‐aa484‐632 plasmids was grown at 37 °C overnight and induced by 1 mM IPTG at 18 °C for 1 h. The cells were collected by centrifugation at 1000 g and fragmented by sonication in GST‐lysis buffer (25 mM Tris‐HCl, pH 8.0, 300 mM NaCl, 5% Glycerol (v/v), 1 mM TCEP). The lysates were centrifuged, and the supernatant was collected, followed loaded onto a pre‐equilibrated PierceTM glutathione column (5 mL, Thermo Fisher, cat 16 110). Then, the column was washed by GST‐lysis buffer (without Glycerol (v/v) and TCEP) and eluted by GST‐elution buffer (25 mM Tris‐HCl, pH 8.0, 300 mM NaCl, 40 mM Reduced Glutathione). Finally, purified proteins were buffer exchanged into PBS.

### GST‐Pull Down

Affinity purified GST‐KAT2B, GST‐KAT2B‐aa484‐632, and His‐EGR1 proteins were subjected to GST pull‐down assay. The conjugation of GST and GST‐Bait‐protein with GSH magnetic beads and the pull‐down assay were performed using BersinBioTM GST‐pull down Kit (BersinBio, Bes3012) according to the manufacturer's instructions. It involves mixing GST‐tagged fusion protein with the target protein to allow binding to GST resin. After washing away unbound proteins, the complex is eluted and analyzed by SDS‐PAGE or Western blot to assess protein interactions.

### In Vitro Lactylation Assays

The design of this experiment referred to Xie et al.^[^ ^70^ ^]^ Briefly, reaction buffer was formulated with HEPES solution (50 mM, pH 7.8), and containing 30 mM KCl, 0.25 mM EDTA, 5.0 mM sodium butyrate, 5.0 mM MgCl_2_, 2.5 mM DTT. The purified EGR1 (100 ng), KAT2B/KAT2B‐aa484‐632 (30ng), and lactyl‐CoA (20µM) were mixed in appropriate amount of reaction buffer, then they were incubated at 30 °C for 3 h. For each assay, reaction products were resolved by 10% SDS‐PAGE and analyzed by immunoblotting.

### RNA Interference

The LDHA and HPSE‐specific siRNAs were designed and synthesized by Shanghai GenePharma Company (Shanghai, China). The MPMVECs were transfected by using CALNPTM RNAi in vitro (D‐Nano Therapeutics, DN001) according to the manufacturer's instructions. siRNA‐targeted sequences were as follows: siRNA‐HPSE (sense 5′‐CCUGAUCUUUGGUCUAAAUTT‐3′, antisense5′‐AUUUAGACCAAAGAUCAGGTT‐3′), siRNA‐LDHA(sense5′‐AGCAAAGACUACUGUGUAACU‐3′, antisense 5′‐UUACACAGUAGUCUUUGCUGG‐3′).

### Lentivirus Infection

Mutant constructs of H3c1‐K18R were generated by using PCR‐based mutagenesis with specific primers (GenePharma). For expression of H3c1WT (NC_000079.7) and H3c1‐K18R, the lentiviral vector LV5 (EF‐1a/GFP&Puro) containing the full‐length sequence of H3c1WT or H3c1‐K18R were transfected into cells according to the manufacturer's protocol. Then, cells were selected based on antibiotic resistance with puromycin (MedChemExpress, Cat.HY‐B1743A).

### Plasmids Transfection

Knockdown of EGR1, CBP and KAT2B were performed using shRNAs from GenePharma Company (Shanghai, China) cloned into pLKO.1, the sequences were listed as follows:CBP‐shRNA,5′‐CCGGTAACTCTGGCCATAGCTTAATCTCGAGATTAAGCTATGGCCAGAGTTATTTTTT‐3′;EGR1‐shRNA,5′‐CCGGGCTGCTTCATCGTCTTCCTCTCTCGAGAGAGGAAGACGATGAAGCAGCTTTTTT‐3′;KAT2B‐shRNA,5′‐CCGGGCAGAGGAGTCCTGTAAATGCCTCGAGGCATTTACAGGACTCCTCTGCTTTTTT‐3′. Overexpressed plasmids pcDNA3.1‐EGR1, pcDNA3.1(+)‐3HA‐KAT2B, pcDNA3.1(+)‐3HA‐CBP (GenePharma) were constructed using the cDNA of EGR1 (NC_000084.7), KAT2B (NC_000083.7) and CBP (NC_000082.7), and transfected with the corresponding empty vector. By using the full‐length KAT2B cDNA as templates, a series of truncated plasmids, including KAT2B‐HA‐ΔPCAF_N (depletion of amino acids: 56–308), KAT2B‐HA‐ΔGNAT (depletion of amino acids: 484–632) and KAT2B‐HA‐ΔBROMO (depletion of amino acids: 721–791), was cloned into pcDNA3.1(+)‐3HA (GenePharma). Mutant constructs of EGR1 (K364R and K402R) were generated by using PCR‐based mutagenesis with specific primers, then inserted into the pcDNA3.1‐N‐3Flag vector (GenePharma). Transfection was achieved using LipofectamineTM 3000 Transfection Reagent (ThermoFisher, L3000015) according to the manufacturers protocol.

### RNA and Stability Assay

For the analysis of RNA stability, MPMVECs were pre‐treated with actinomycin D (2 ug mL^−1^) (Sigma, SBR00013) for 1 h, followed stimulated by lactate (8 mM) 7 h. Then, total cellular RNA was extracted and RNA levels were quantified by RT‐qPCR.

### Statistical Analysis

Statistical analysis was done using GraphPad Prism 9.0, SPSS software (SPSS version 24.0; SPSS Inc, IL), STATA 15.1 (College Station, Texas) and R 3.6.2 (Chicago, Illinois) software. Independent‐sample Student's t‐test was applied for comparison between two groups. To compare three or more experimental groups, one‐way and two‐way analyses of variance (ANOVA) were performed. Survival differences were determined using the Kaplan‐Meier method and the log‐rank test. In the figures, error bars reflect the mean±standard deviation (SD). The investigators were blinded to the group allocation during the experiment and data collection. For clinical data, this work first performed a normality test (Agostino tests), followed by a descriptive analysis of the data. Continuous variables were expressed as mean (standard deviation) while non‐parametric variables were expressed as the median (interquartile ranges, IQR) and were compared using the one‐way ANOVA test or non‐parametric Kruskal‐Wallis test. The categorical variables are expressed as a frequency (percentage) and were compared using the *X* ^2^ or rank‐sum tests. Correlation and linear regression were calculated using Spearmans method for non‐parametric data, or Pearsons test for the parametric. This work also conducted the dose‐response association using the restricted cubic splines model with five knots located at the 5, 35, 50, 65, and 95th percentiles of the overall distribution for exposure based on the multivariate logistic regression model. *p* < 0.05 was considered statistically significant.

## Conflict of Interest

The authors declare no conflict of interest.

## Author Contributions

Q.Y., G.S., and L.F conceptualized and designed this study. Z.L., P.F., S.L, and D.L., conducted the experiments and revision. Z.L, J.Z., X.W, and J.P analyzed the data, produced the figures, and composed the manuscript. H.J help to performed SEM&TEM assay. Q.Y supervised the experiments. Z.L., P.F., and S.L contributed equally to this study. All authors approved the final version of the manuscript.

## Supporting information



Supporting Information

Supporting Table

## Data Availability

The data that support the findings of this study are available from the corresponding author upon reasonable request.
