# Supplementary material for: Lactylation of Histone H3k18 and Egr1 Promotes Endothelial Glycocalyx Degradation in Sepsis‐Induced Acute Lung Injury
Source: Adv Sci (Weinh). 2024 Dec 25;12(7):2407064. doi: 10.1002/advs.202407064 (PMC11831459; doi:10.1002/advs.202407064)
Supplement: Supplementary file 1 — Supporting Information [file ADVS-12-2407064-s002.docx]

*Supplementary Materials for*

**LACTYLATION OF HISTONE H3K18 AND EGR1 PROMOTES ENDOTHELIAL GLYCOCALYX DEGRADATION IN SEPSIS-INDUCED ACUTE LUNG INJURY**

Zongqing Lu, Pu Fang, Shuai Li, *et al.*

*Corresponding author: Qinghai You, amormor@126.com;

Gengyun Sun, sungengy@126.com;

Lin Fu, fulindev@126.com

**Supplementary Figures and Tables**

**
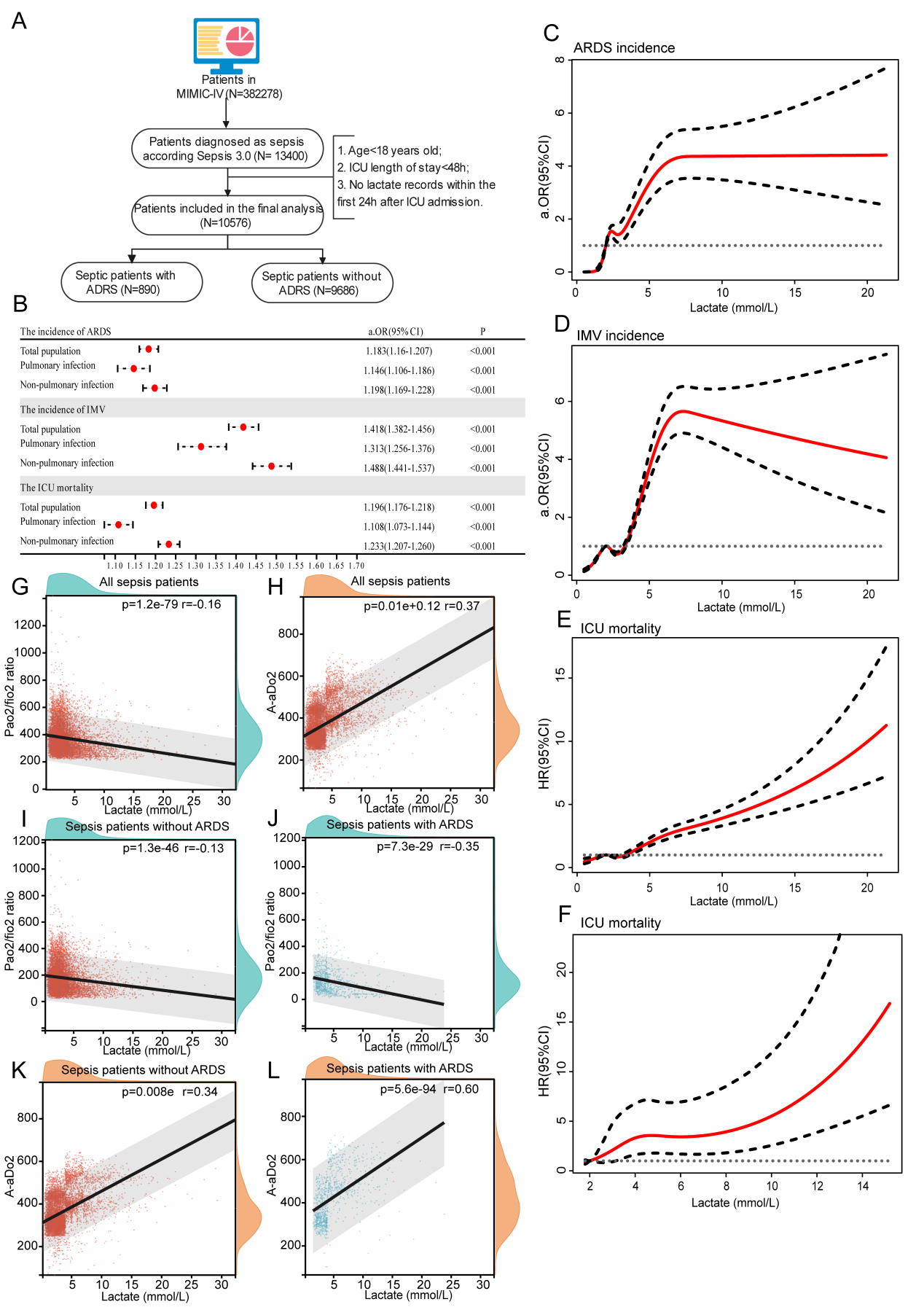
**

Figure.S1: An increased lactate level is corrected with a high incidence of ARDS and poor prognosis in sepsis patients.

A) Flow chart showing the sepsis patients screening process in MIMIC-IV database.

1. Forest plot showing the a.ORs (95%CI) for the ARDS, invasive mechanical ventilation incidence (IMV), and ICU death risk across different initial blood lactate levels based on multivariable logistic regression analysis.

(C-E) Restricted cubic spline of the ARSD, IMV incidence and ICU mortality in sepsis patients.

F) Restricted cubic spline of the ICU mortality in sepsis associated ARSD patients.

G-H) Correlation analysis of blood lactate levels with Pao_2_/Fio_2_ and A-aDo_2_ index in all sepsis patients.

I-J) Correlation analysis of blood lactate levels with Pao_2_/Fio_2_ index in the subgroup of sepsis patients with or without ARDS.

K-L) Correlation analysis of blood lactate levels with A-aDo_2_ index in the subgroup of sepsis patients with or without ARDS.


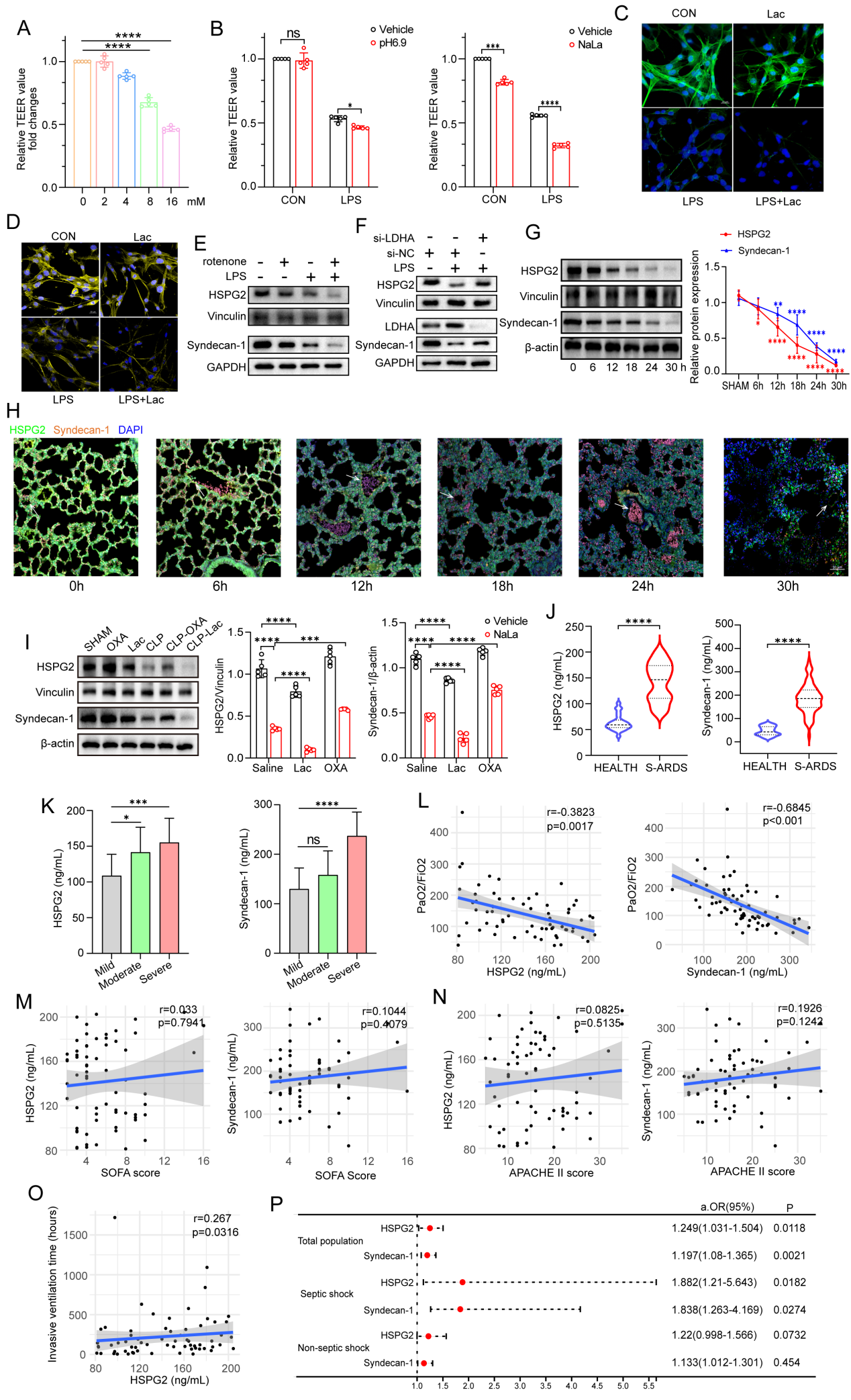


Figure S2: Lactate exacerbates glycocalyx degradation on MPMVECs and in vivo during sepsis.

1. The effect of different concentrations of lactate on the TEER of MPMVECs (n=5).
2. The effect of acidic condition or NaLa on the TEER of MPMVECs (n=5).

C) Representative immunofluorescent staining images of HSPG2 (green), syndecan-1 (yellow), and nuclei (DAPI, blue) in MPMVECs treated with lactate, LPS, and both. Scale bar, 20μm.

D) Representative immunofluorescent staining images of syndecan-1 (yellow), and nuclei (DAPI, blue) in MPMVECs treated with lactate, LPS, and LPS+lactate. Scale bar, 20μm.

E) MPMVECs were treated with rotenone (5nM) for 24h after LPS stimulation, the expression levels of HSPG2 and syndecan-1 were detected by Western blot.

F) MPMVECs were transfected with siRNAs for LDHA and scramble control siRNA before LPS stimulation for 12 h, the expression levels of HSPG2 and syndecan-1 were detected by Western blot.

G) The expression levels of HSPG2 and syndecan-1 in lung tissue at different times (hours) after CLP surgery (n=5 per group).

H) Representative immunofluorescent staining images of HSPG2 (green) and syndecan-1 (yellow) in endothelial surface of the lung tissues. Nuclei were stained with DAPI (blue). Scale bar, 50μm.

I) The effect of OXA and lactate on the expression of HSPG2 and syndecan-1 after Sham or CLP surgery (n=5 per group).

J) Comparation of serum HSPG2 and syndecan-1 concentration between health individuals and S-ARDS patients.

K) The serum HSPG2 and syndecan-1 concentration in mild, moderate, and severe S-ARDS patients.

L) Correlation analysis of serum HSPG2, syndecan-1 and PaO_2_/FiO_2_ levels in S-ARDS patients.

M) Correlation analysis of serum HSPG2, syndecan-1 and SOFA score in S-ARDS patients.

N) Correlation analysis of serum HSPG2, syndecan-1 and APACHE II score in S-ARDS patients.

O) Correlation analysis of serum HSPG2 and invasive ventilation duration in S-ARDS patients.

P) a.ORs and 95%CI in 28-day mortality per 10ng/ml increase in the serum concentration of HSPG2 and syndecan-1.

All data were represented as the means±SD, **P* < 0.05, ***P* < 0.01, ****P* < 0.001, and *****P* < 0.0001; ns, not significant.


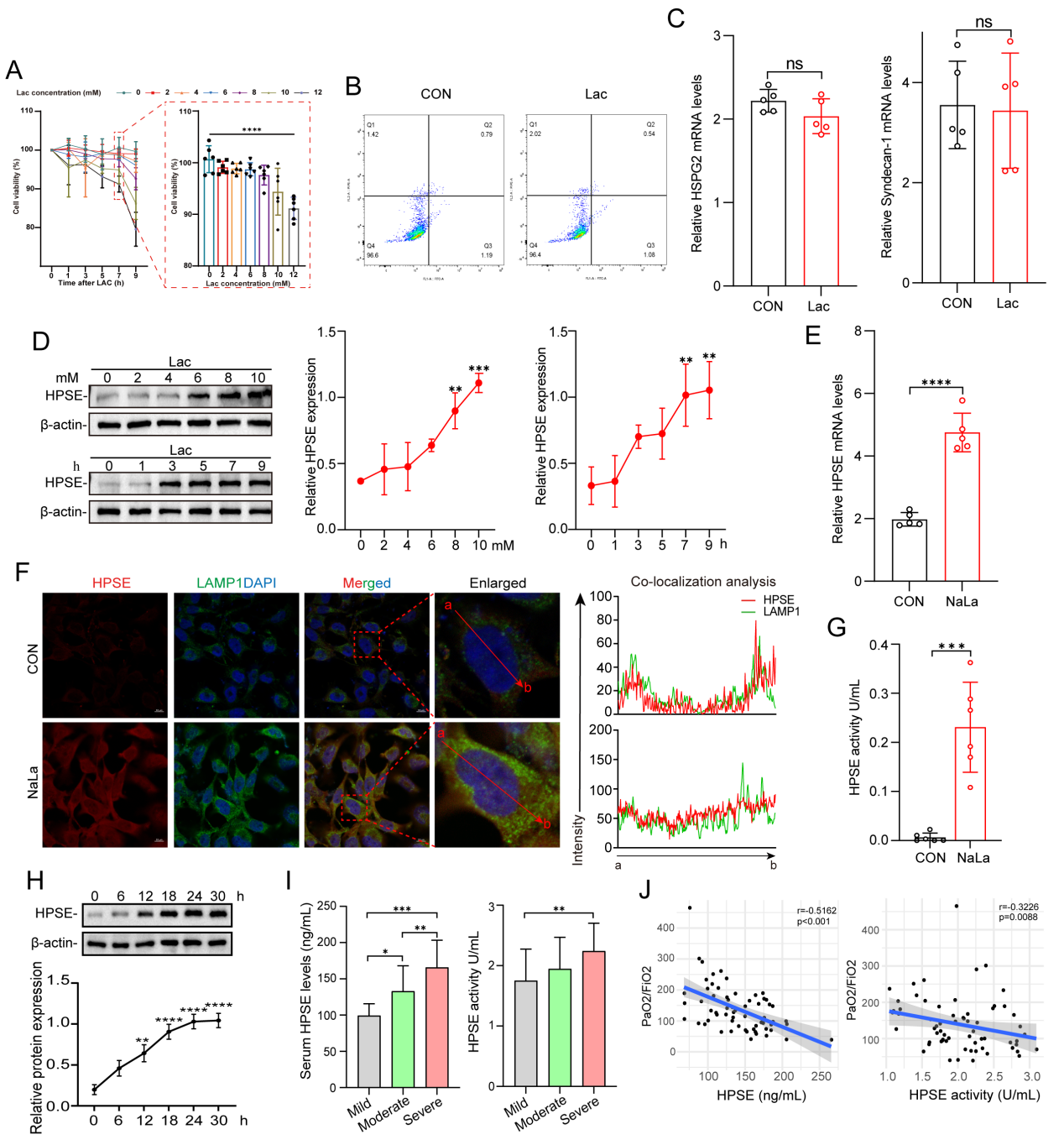


Figure S3: Lactate promotes glycocalyx degradation by increasing the expression of HPSE.

1. The effects of different concentrations and stimulation times of lactate on the MPMVECs viability (n=6).
2. MPMVECs were stimulated with lactate (8 mM) for 7 hours. Cell death and apoptosis were assessed by flowcytometry using anti-PI and anti-Annexin V (FITC) antibodies.
3. RT-qPCR was used to detect the HSPG2 and syndecan-1 mRNA level of MPMVECs after lactate (8mM for 7h) treatment, respectively (n=5).
4. The effects of different concentrations and stimulation times of lactate on the HPSE expression levels in MPMVECs (n=5).
5. RT-qPCR was used to detect the HPSE mRNA level of MPMVECs after NaLa (8mM for 7h) treatment (n=5).
6. MPMVECs were treated with NaLa (8mM for 7h). HPSE (red) and LAMP1 (green) co-localization was examined by confocal microscope (scale bar, 10um). Nucleus was indicated by DAPI (blue) staining. Co-localization analysis was performed by ImageJ. Scale bar, 10μm.
7. The levels of HPSE activity in MPMVECs after NaLa treatment (8mM for 7h) (n=6).
8. The effects of different stimulation times of lactate on the expression of HSPG2 and syndecan-1 in MPMVECs (n=3).
9. The serum HPSE concentration and activity in mild, moderate, and severe S-ARDS patients.
10. Correlation analysis of serum HPSE concentration and activity with Pao_2_/Fio_2_ index in S-ARDS patients.

All data were represented as the means±SD, **P* < 0.05, ***P* < 0.01, ****P* < 0.001, and *****P* < 0.0001; ns, not significant.


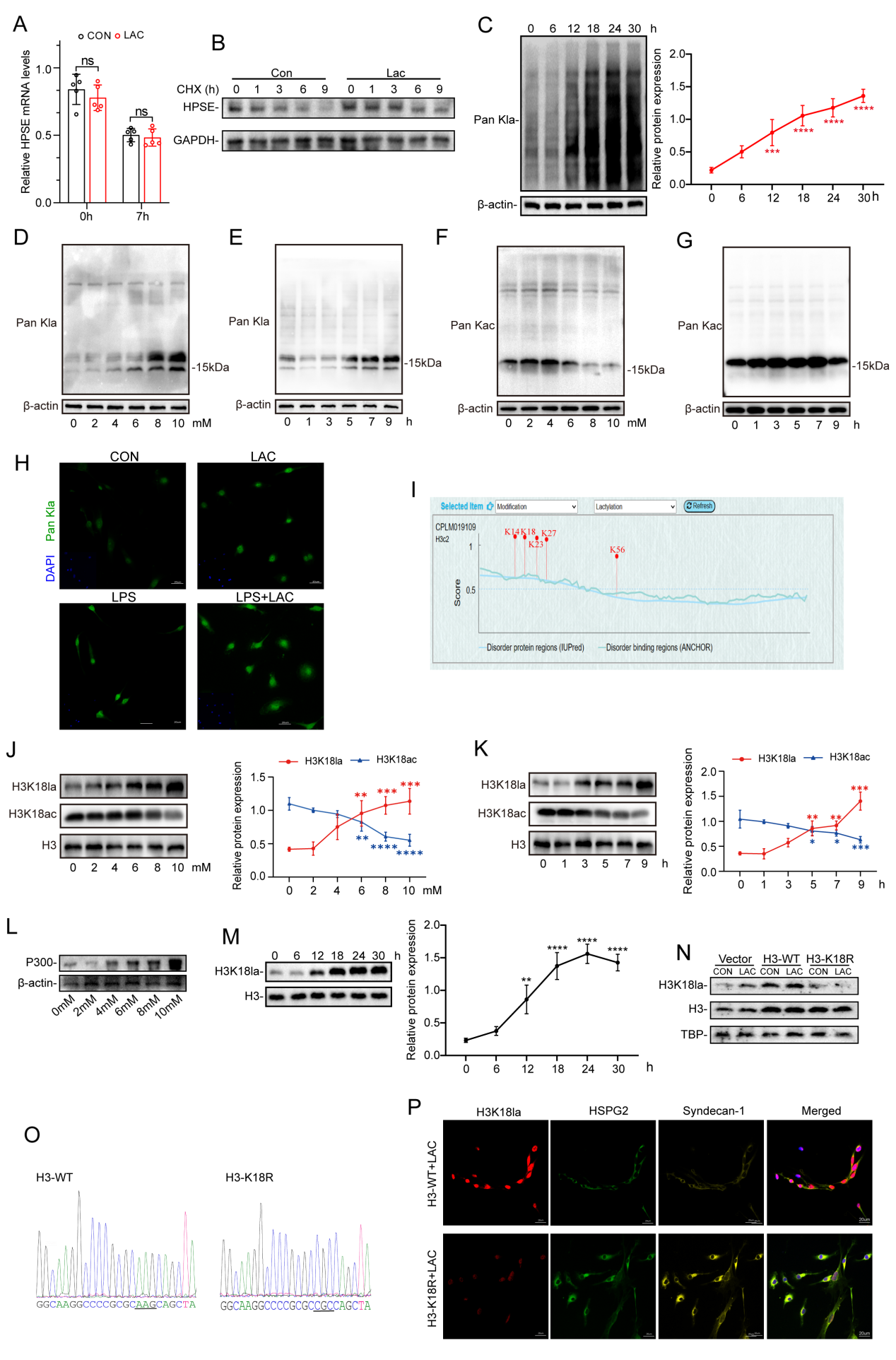


Figure S4: Global lactylation and H3K18la levels in the lung are increased during sepsis.

1. MPMVECs were pre-treated with 2ug/mL actinomycin D for 1h and further stimulated with 8mM lactate for 7h or not. The mRNA of HPSE was measured by RT-qPCR (n=5).
2. Western blotting analysis of HPSE in MPMVECs treated with 0.1 mg/mL cyclohexane for indicated time.

C) The expression levels of Pan Kla in lung tissue at different times (hours) after CLP surgery (n=5 per group).

D) The effect of different concentrations of lactate on the Pan Kla levels in MPMVECs.

E) The effects of different treatment times of lactate on the Pan Kla levels in MPMVECs.

F) The effect of different concentrations of lactate on the Pan Kac levels in MPMVECs.

G) The effects of different treatment times of lactate on the Pan Kac levels in MPMVECs.

H) Immunofluorescence of H3K18la (green) in MPMVECs after treatment with LPS (12ug/mL for 12h), lactate (8mM for 7h), or both. Nuclei were stained with DAPI (blue). Scale bar, 20μm.

I) Lactylation scores for Histone H3 sites were predicted on CPLM (http://cplm.biocuckoo.cn/index.php).

J) The effect of different concentrations of lactate on the H3K18la and H3K18ac levels in MPMVECs (n=3).

K) The effect of different treatment times of lactate on the H3K18la and H3K18ac levels in MPMVECs (n=3)

L) Expression of P300 in MPMVECs after the stimulation of different concentrations of lactate.

M) The expression levels of H3K18la in lung tissue at different times (hours) after CLP surgery (n=5 per group).

N) K18R site mutation or wild-type histone H3 overexpressed MPMVECs were constructed respectively through lentiviral vector. H3K18la levels were analyzed by western blotting.

O) Chromatographs of Sanger sequencing of genes H3-WT and H3-K18R.

P) Representative immunofluorescent staining images of H3K18la (red), HSPG2 (green), syndecan-1 (yellow), and nuclei (DAPI, blue) in K18R site mutation or wild-type histone H3 overexpressed MPMVECs after 8 mM lactate treatment for 7h. Scale bar, 20μm.

All data were represented as the means±SD, **P* < 0.05, ***P* < 0.01, ****P* < 0.001, and *****P* < 0.0001; ns, not significant.


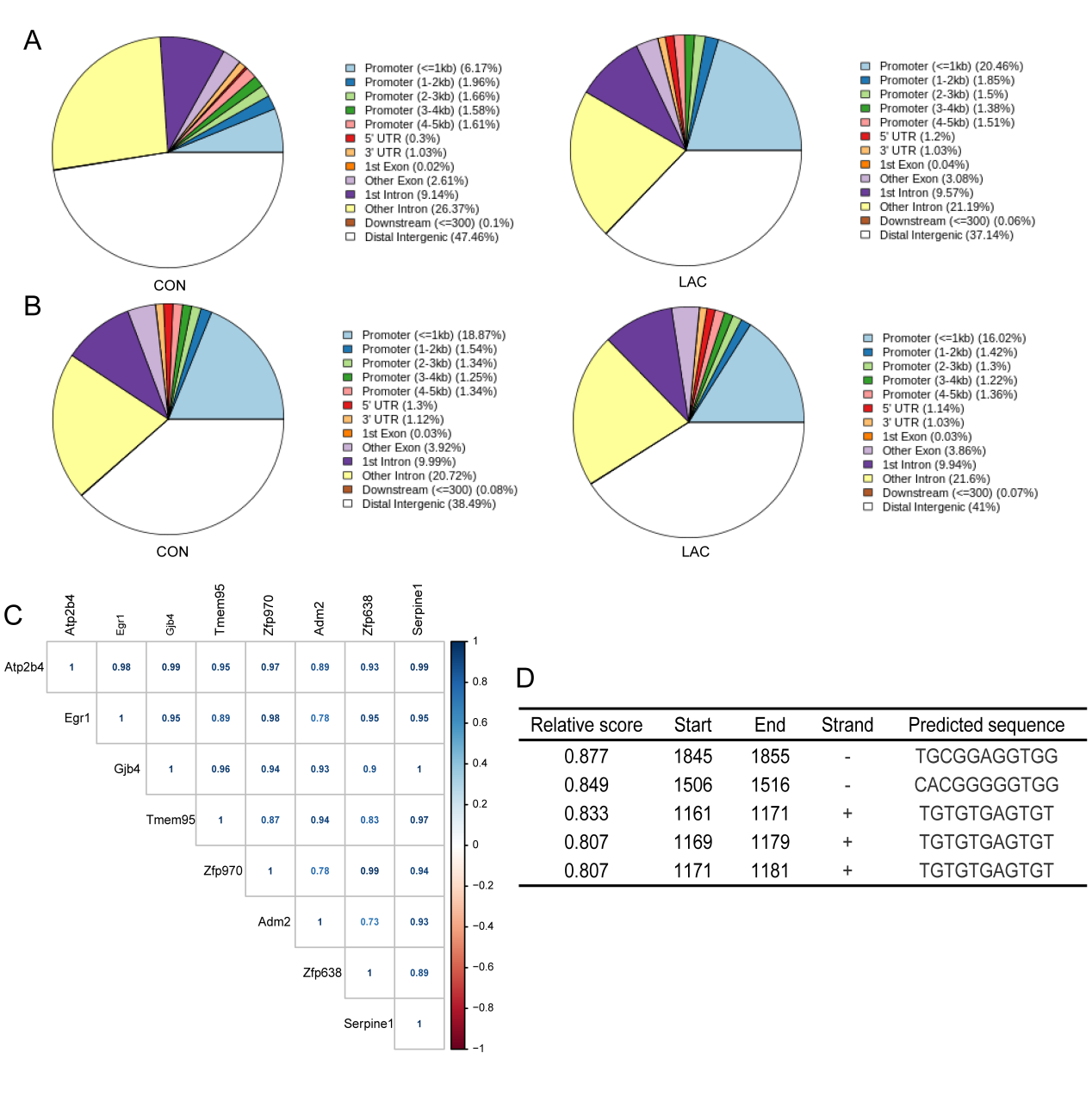


Figure S5: H3K18la enhances the expression of HPSE by increasing the activation of EGR1

1. Pie chart showing the distribution of H3K18la and B) H3K18ac at annotated genomic regions in MPMVECs after 8mM lactate stimulation for 7h.

C) Correlation heatmap of genes with increased transcription levels and enriched H3K18la in the promoter region.

D) JASPAR-predicted binding sites of EGR1 within the HPSE gene regulatory region with location, strand and relative binding score (with 80% cutoff).


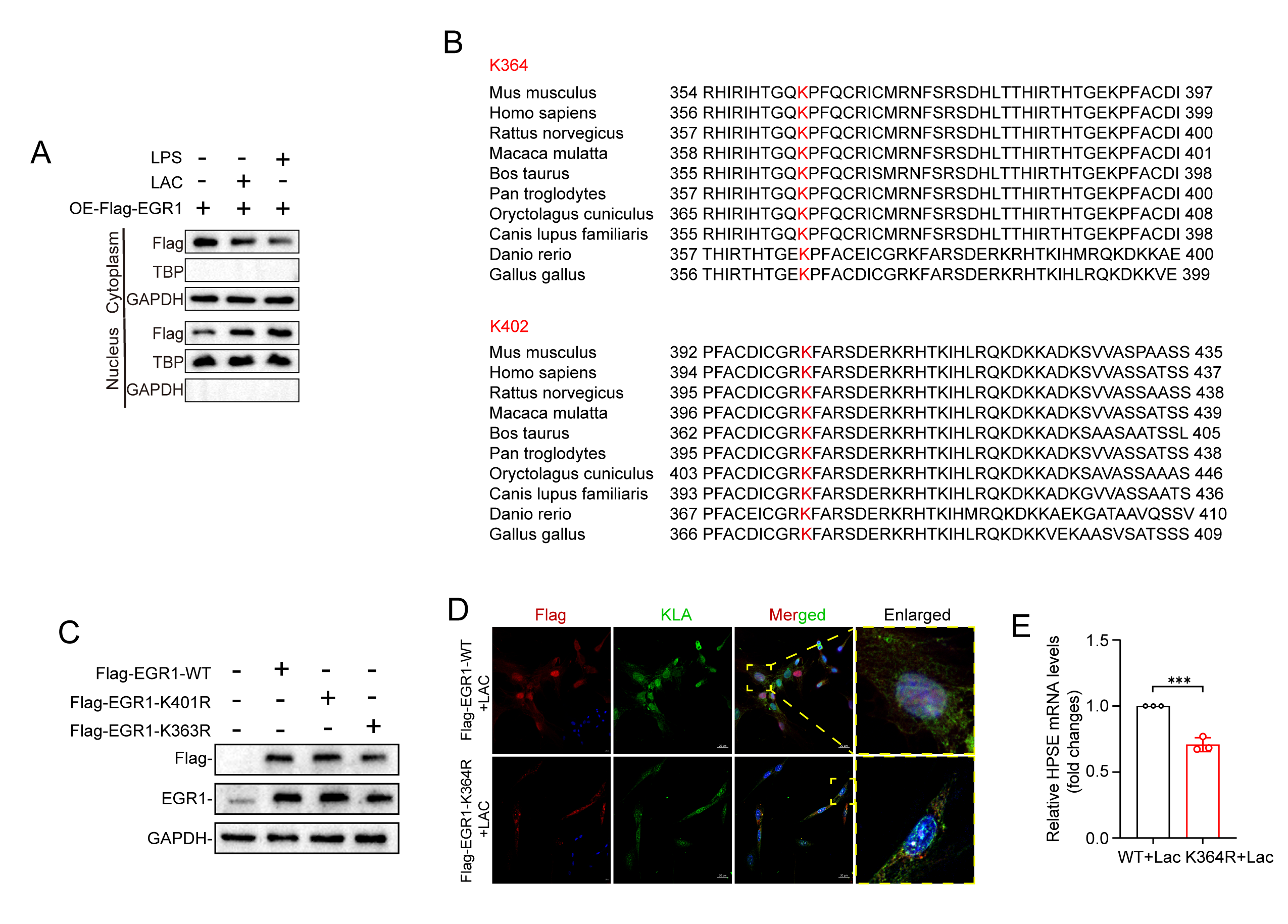


Figure S6: Lactate increases EGR1 binding to Importin-α via K364la, in turn facilitating EGR1 nuclear localization.

1. Effect of LPS and lactate on nuclear translocation of exogenous Flag-EGR1 in MPMVECs.
2. The K364 and K402 sites of EGR1 are conserved.
3. K364R, K402R, or wild-type Flag-EGR1 overexpressed MPMVECs were constructed respectively through overexpression plasmid.
4. After treatment with lactate (8mM for 7h), immunofluorescence of Flag-EGR1 protein (red) and Pan Kla (green) in MPMVECs overexpressing Flag-EGR1 or Flag-EGR1-K364R mutant. Nuclei were stained with DAPI (blue). Scale bar, 20μm.
5. RT-qPCR was used to detect the HPSE mRNA level in MPMVECs overexpressing Flag-EGR1 or Flag-EGR1-K364R mutant after lactate (8mM for 7h) treatment (n=3).

All data were represented as the means±SD, **P* < 0.05, ***P* < 0.01, ****P* < 0.001, and *****P* < 0.0001; ns, not significant.


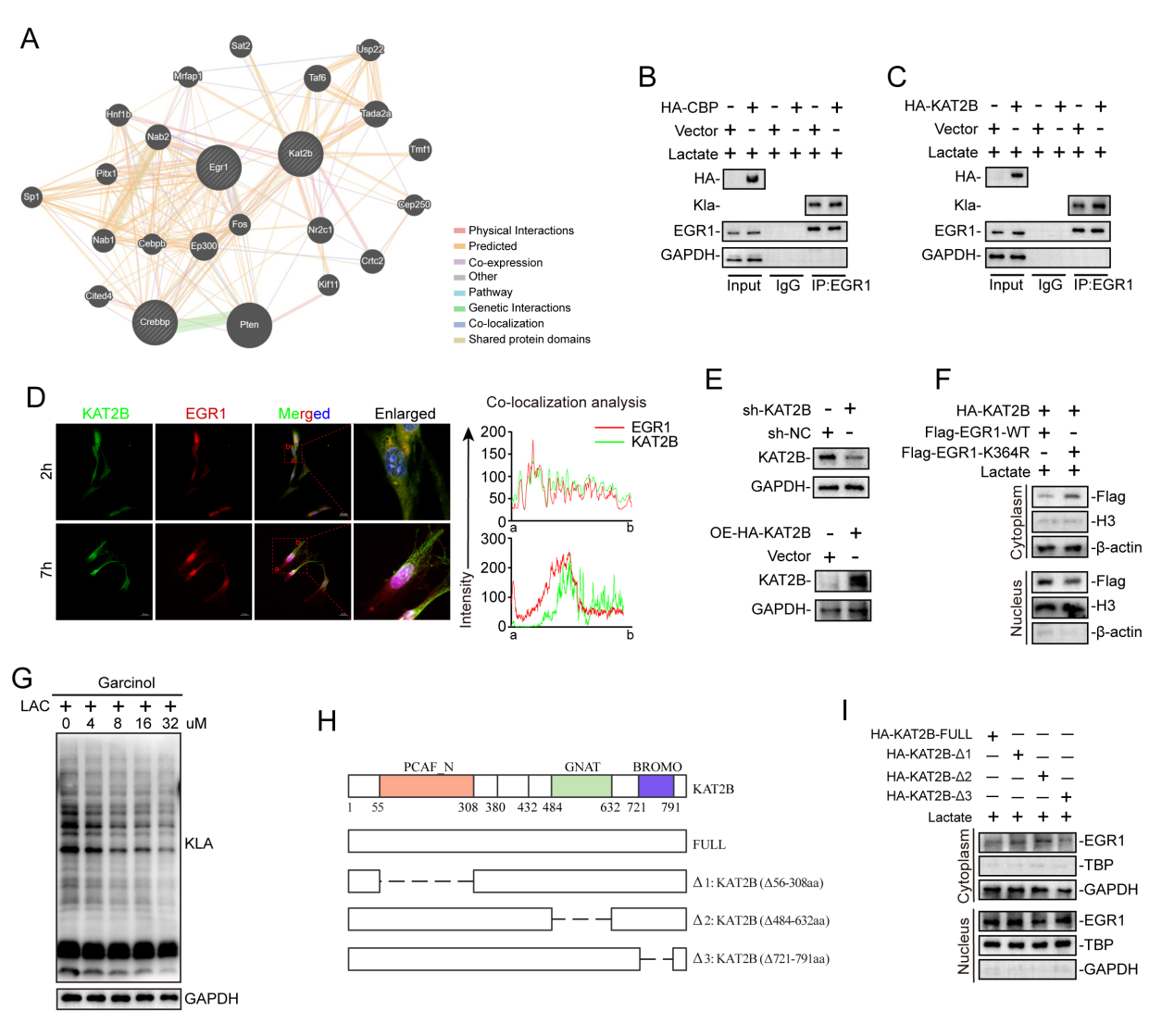


Figure S7: Identification of KAT2B as an EGR1 lactyltransferase.

1. Protein-protein interaction networks of EGR1, CBP and KAT2B were analyzed by Genemania.
2. Lactylation levels of EGR1 in control vector or HA-tagged CBP overexpressed MPMVECs after lactate stimulation (8mM).
3. Lactylation levels of EGR1 in control vector or HA-tagged KAT2B overexpressed MPMVECs after lactate stimulation (8mM).
4. After treatment with lactate (8mM) for indicated time, KAT2B (green) and EGR1 (red) co-localization was examined by confocal microscope (scale bar, 20um). Nucleus was indicated by DAPI (blue) staining. Co-localization analysis was performed by ImageJ.
5. Overexpression or knockdown of KAT2B in MPMVECs was confirmed with Western blot assay.
6. After overexpressing with Flag-EGR1-WT or Flag-EGR1-K364R, cytoplasmic and nuclear Flag-EGR1 protein levels was measured in HA-tagged KAT2B-overexpressing MPMVECs.
7. After treatment with different concentration of Garcinol, global Kla levels of MPMVECs in the presence of lactate (8mM).
8. Diagram of the different domains of the KAT2B constructs and truncated plasmids.
9. After treatment with lactate (8mM for 7H), cytoplasmic and nuclear EGR1 expression was measured in different HA-tagged KAT2B truncations-overexpressed MPMVECs.

All data were represented as the means±SD, **P* < 0.05, ***P* < 0.01, ****P* < 0.001, and *****P* < 0.0001; ns, not significant.


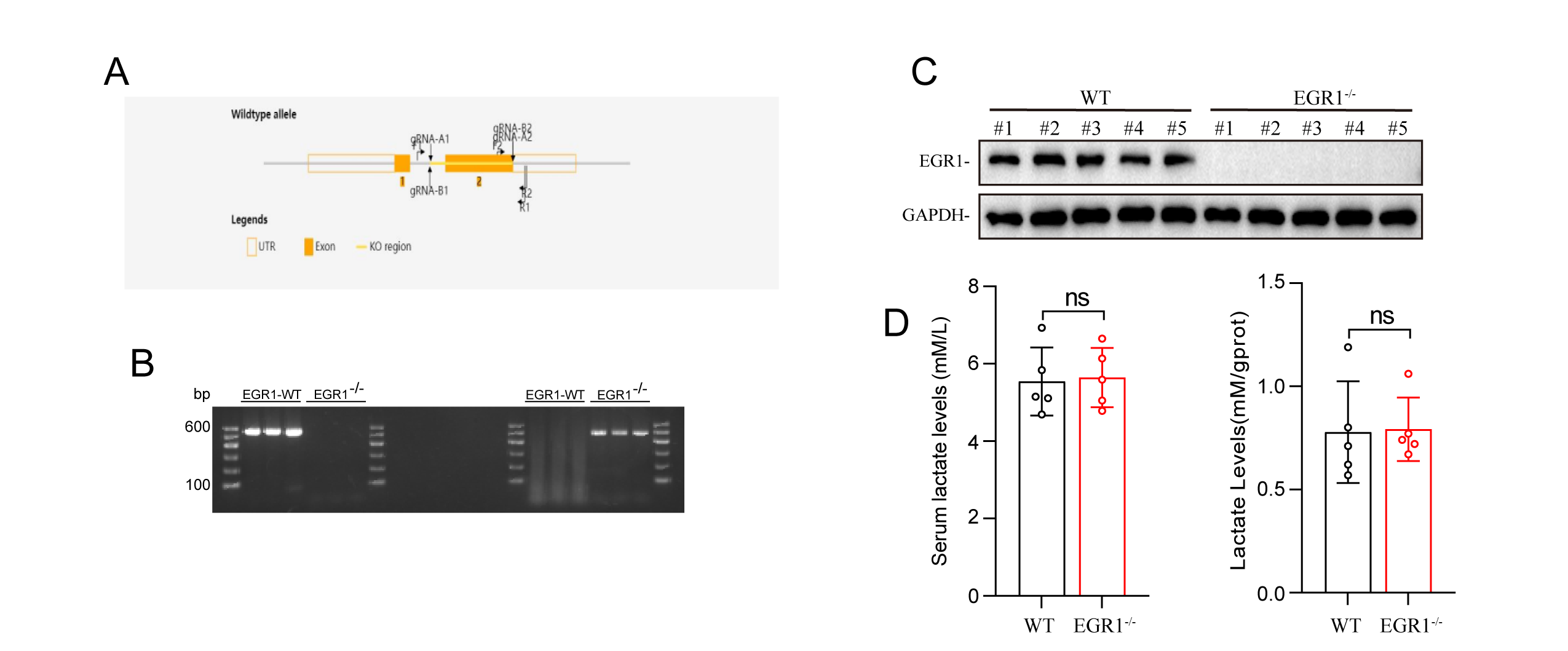


Figure S8: EGR1 knockout attenuates glycocalyx degradation and ALI in mice with polymicrobial sepsis.

1. Diagram of the constructed model of EGR1 knockout (EGR1^-/-^) mice.
2. Mouse genotypes were identified by PCR using the two pair of primers. The PCR product length was 566 bp for wild-type (WT) mice and 476 bp for EGR1^-/-^ mice.
3. Expression of EGR1 protein in lung tissue lysate of EGR1-WT or EGR1^-/-^ mice.
4. Serum and lung tissue lactate concentration of EGR1-WT or EGR1-/- mice after CLP surgery.

Table S1 The characteristics of included patients when first ICU admission in MIMIC-IV database.

| Variables | Total (n = 10576) | Sepsis patient without ARDS  (n = 9686) | Sepsis patient with ARDS  (n = 890) | P value |
| --- | --- | --- | --- | --- |
| Lactate | 2.7 (2, 3.7) | 2.64 (1.9, 3.6) | 3.78 (2.7, 5.8) | < 0.001 |
| pH | 7.31 (7.25, 7.36) | 7.31 (7.26, 7.36) | 7.22 (7.13, 7.29) | < 0.001 |
| So2 | 91 (80.94, 95) | 91 (81, 95) | 90.26 (80, 95) | 0.359 |
| Po2 | 75 (46, 100) | 76 (46, 100.33) | 65 (45, 86) | < 0.001 |
| Pco2 | 47.73 (41, 53) | 47.49 (41, 52) | 50 (43, 60) | < 0.001 |
| A-ado2 | 420.74 (396.67, 483.86) | 420.23 (396.56, 479.08) | 435 (399, 522.45) | < 0.001 |
| Pao2/Fio2 | 172 (106, 215.69) | 175 (108.03, 216.68) | 128.33 (80, 190) | < 0.001 |
| Spo2 | 93 (90, 95) | 93 (90, 95) | 92 (89, 95) | 0.202 |
| Baseexcess | -3 (-6, 0) | -3 (-6, 0) | -5 (-10, -1) | < 0.001 |
| Hematocrit | 29.2 (24.9, 34) | 29.1 (24.9, 33.9) | 30.9 (25.1, 35.6) | < 0.001 |
| Hemoglobin | 9.7 (8.2, 11.3) | 9.7 (8.2, 11.2) | 10 (8.3, 11.8) | < 0.001 |
| Platelets | 159 (108, 224) | 159 (109, 225) | 158 (98, 218) | 0.02 |
| WBC count | 14.3 (10.3, 19.5) | 14.3 (10.3, 19.4) | 15.2 (11.1, 20.8) | < 0.001 |
| Albumin | 3.2 (2.9, 3.37) | 3.2 (2.9, 3.37) | 3.16 (2.72, 3.38) | 0.002 |
| Aniongap | 13 (11, 15) | 13 (11, 15) | 13 (11, 15) | 0.016 |
| Bicarbonate | 21 (18, 24) | 21 (18, 24) | 20 (16, 23) | < 0.001 |
| BUN | 24 (16, 40) | 24 (16, 41) | 23 (16, 35) | 0.009 |
| CRE | 1.2 (0.8, 1.9) | 1.2 (0.8, 1.9) | 1.2 (0.8, 1.8) | 0.357 |
| Glucose | 113 (95, 136) | 113 (95, 136) | 115 (96, 137) | 0.177 |
| Basophils | 0.03 (0.02, 0.04) | 0.03 (0.02, 0.04) | 0.03 (0.01, 0.04) | 0.003 |
| Eosinophils | 0.09 (0.02, 0.15) | 0.09 (0.02, 0.15) | 0.08 (0.01, 0.13) | < 0.001 |
| Lymphocytes | 1.43 (0.86, 1.98) | 1.43 (0.87, 1.98) | 1.36 (0.81, 1.9) | 0.009 |
| Monocytes | 0.61 (0.44, 0.84) | 0.61 (0.44, 0.83) | 0.63 (0.4, 0.86) | 0.54 |
| Neutrophils | 10.56 (8.54, 13.62) | 10.51 (8.53, 13.55) | 11.18 (8.68, 14.9) | < 0.001 |
| INR | 1.4 (1.2, 1.7) | 1.4 (1.2, 1.7) | 1.35 (1.1, 1.7) | 0.003 |
| PT | 15.1 (13.3, 18.2) | 15.1 (13.3, 18.2) | 14.68 (12.5, 18.4) | < 0.001 |
| PTT | 34.5 (28.9, 46) | 34.4 (28.9, 45.9) | 34.9 (29.6, 48.1) | 0.019 |
| ALT | 59 (25, 95.94) | 58.92 (25, 95) | 62 (25, 112.51) | 0.026 |
| ALP | 86.43 (66, 124) | 86.92 (66.11, 124.7) | 84 (62, 117.94) | < 0.001 |
| AST | 93.87 (38, 149.82) | 93.15 (38, 148) | 102.92 (39, 178.03) | 0.002 |
| SBP | 112.41 (104.39, 123.21) | 112.48 (104.38, 123.25) | 111.62 (104.48, 122.63) | 0.419 |
| DBP | 60.09 (54.27, 66.82) | 60 (54.14, 66.8) | 61.17 (55.96, 67.02) | < 0.001 |
| MAP | 75.05 (69.72, 81.8) | 75.03 (69.69, 81.76) | 75.48 (70.12, 82.29) | 0.067 |
| Heart_rate | 86.36 (76.23, 98.67) | 86 (76.04, 98.2) | 90.88 (79, 103.91) | < 0.001 |
| Resp_rate | 19.25 (16.92, 22.35) | 19.17 (16.85, 22.23) | 20.65 (17.9, 23.78) | < 0.001 |
| Temperature | 36.92 (36.64, 37.32) | 36.89 (36.62, 37.24) | 37.57 (37.26, 37.96) | < 0.001 |
| Age | 67.94 (56.27, 79.05) | 68.46 (56.88, 79.37) | 61 (48.54, 71.7) | < 0.001 |
| Gender, n (%) |  |  |  | 0.981 |
| M | 7695 (57) | 7144 (57) | 551 (57) |  |
| F | 5705 (43) | 5295 (43) | 410 (43) |  |
| SOFA score | 3 (2, 5) | 3 (2, 4.5) | 3 (2, 5) | < 0.001 |
| IMV, n (%) |  |  |  | < 0.001 |
| No | 4858 (36) | 4857 (39) | 1 (0) |  |
| Yes | 8542 (64) | 7582 (61) | 960 (100) |  |
| Mortality_icu, n (%) |  |  |  | < 0.001 |
| No | 11750 (88) | 10968 (88) | 782 (81) |  |
| Yes | 1650 (12) | 1471 (12) | 179 (19) |  |
| Mortality_7day, n (%) |  |  |  | 0.003 |
| No | 12140 (91) | 11296 (91) | 844 (88) |  |
| Yes | 1260 (9) | 1143 (9) | 117 (12) |  |
| Mortality_28day, n (%) |  |  |  | 0.003 |
| No | 10637 (79) | 9910 (80) | 727 (76) |  |
| Yes | 2763 (21) | 2529 (20) | 234 (24) |  |
| Mortality_hospital, n (%) |  |  |  | < 0.001 |
| No | 11043 (82) | 10303 (83) | 740 (77) |  |
| Yes | 2357 (18) | 2136 (17) | 221 (23) |  |
| Respiratory_infections, n (%) |  |  |  | < 0.001 |
| No | 9252 (69) | 8709 (70) | 543 (57) |  |
| Yes | 4148 (31) | 3730 (30) | 418 (43) |  |
| ICU stay duraiton (days) | 4.21 (2.86, 7.72) | 4.06 (2.8, 7.14) | 8.09 (4.72, 13.49) | < 0.001 |

Abbreviations: WBC, white blood cells, SBP, systolic blood pressure, DBP, diastolic blood pressure, MAP, mean arterial pressure,

BUN,blood urea nitrogen, CRE, creatinine, IMV, invasive mechanical ventilation.

Table S2 The characteristics of sepsis-related ARDS patients with different severity.

| Variables | Total (n = 65) | Mild (n = 11) | Moderate (n = 29) | Severe (n = 25) | P value |
| --- | --- | --- | --- | --- | --- |
| Gender, n (%) |  |  |  |  | 1 |
| M | 46 (71) | 8 (73) | 20 (69) | 18 (72) |  |
| F | 19 (29) | 3 (27) | 9 (31) | 7 (28) |  |
| Age | 75 (67, 81) | 80 (68, 81) | 75 (67, 80) | 75 (59, 81) | 0.916 |
| Pco2 | 38.7 (31.2, 52.4) | 36.6 (32.85, 43.3) | 44.5 (34.3, 56) | 37.5 (27.9, 50.9) | 0.289 |
| Sao2 | 93 (87.6, 96) | 98 (93.7, 99.5) | 94 (89, 96) | 90 (78, 93) | 0.001 |
| Po2 | 64.1 (52.4, 81) | 100 (75.9, 107.5) | 68.7 (55, 81) | 58 (41, 65) | <0.001 |
| Po2/Fio2 | 126 (89, 176) | 237 (212.55, 275.7) | 147.6 (126, 168) | 74 (64.2, 92) | <0.001 |
| PEEP | 5(3, 6) | 3(0,4) | 5(3, 5) | 5(3, 8.25) | 0.085 |
| SOFA score | 5 (3, 7) | 4 (3.5, 5.5) | 5 (3, 9) | 6 (4, 7) | 0.769 |
| APACHEII score | 15 (12, 20) | 14 (12, 16) | 14 (10, 23) | 16 (12, 20) | 0.586 |
| Hypertension, n(%) |  |  |  |  | 0.702 |
| No | 41 (63) | 6 (55) | 18 (62) | 17 (68) |  |
| Yes | 24 (37) | 5 (45) | 11 (38) | 8 (32) |  |
| Diabete, n(%) |  |  |  |  | 0.924 |
| No | 51 (78) | 9 (82) | 22 (76) | 20 (80) |  |
| Yes | 14 (22) | 2 (18) | 7 (24) | 5 (20) |  |
| Cerebrovascular, n(%) |  |  |  |  | 0.038 |
| No | 55 (85) | 7 (64) | 24 (83) | 24 (96) |  |
| Yes | 10 (15) | 4 (36) | 5 (17) | 1 (4) |  |
| Cancer, n(%) |  |  |  |  | 0.105 |
| No | 54 (83) | 11 (100) | 25 (86) | 18 (72) |  |
| Yes | 11 (17) | 0 (0) | 4 (14) | 7 (28) |  |
| Heart_rate | 110 (85, 120) | 116 (84, 120) | 115 (83, 122) | 106 (96, 119) | 0.758 |
| SBP | 116.95 ± 21.84 | 117.82 ± 26.12 | 117.97 ± 17.67 | 115.4 ± 24.89 | 0.905 |
| DBP | 68.34 ± 12.16 | 66.36 ± 11.25 | 69.14 ± 11.99 | 68.28 ± 13.1 | 0.817 |
| Resp_rate | 22 (20, 28) | 22 (21, 30) | 22 (20, 26) | 24 (20, 27) | 0.639 |
| Temperature | 37 (36.6, 37.5) | 37 (36.6, 37.45) | 37 (36.8, 37.8) | 37 (36.6, 37.3) | 0.787 |
| WBC count | 10.63 (7.76, 14.3) | 10.12 (7.96, 12.42) | 10.32 (7.81, 14.16) | 12.34 (7.77, 14.73) | 0.839 |
| Neutrophil count | 8.75 (7.07, 10.98) | 8.01 (7.3, 9.58) | 8.63 (6.06, 10.56) | 10.02 (7.64, 13.66) | 0.312 |
| Lymphocyte count | 0.62 (0.37, 0.96) | 0.79 (0.58, 1.44) | 0.59 (0.44, 0.79) | 0.51 (0.23, 0.95) | 0.155 |
| Monocytes count | 0.39 (0.22, 0.53) | 0.64 (0.53, 0.9) | 0.39 (0.22, 0.5) | 0.28 (0.12, 0.44) | 0.002 |
| Hemoglobin | 110.12 ± 24.5 | 105.4 ± 21.09 | 109.34 ± 22.87 | 112.92 ± 27.94 | 0.702 |
| Platelet | 187 (116, 254) | 207 (187.5, 352.5) | 170 (98, 220) | 187 (139, 254) | 0.103 |
| CRP | 93.17 (36.22, 160.85) | 58.2 (39.55, 118.93) | 96.63 (24.21, 144.45) | 79.35 (38.25, 180.23) | 0.889 |
| PCT | 0.57 (0.13, 2.46) | 0.19 (0.1, 0.64) | 0.59 (0.09, 2.46) | 0.81 (0.22, 2.75) | 0.362 |
| PT | 12.7 (11.7, 14.4) | 12.2 (11.8, 13.2) | 12.7 (11.9, 14.9) | 12.8 (11.7, 14.8) | 0.815 |
| APTT | 30.2 (27.9, 37.3) | 30.2 (29.6, 33.55) | 31.3 (28.2, 39.5) | 29.5 (27.1, 35.2) | 0.632 |
| Fibrinogen | 4.22 (2.7, 5.54) | 3.84 (2.53, 4.82) | 4.19 (2.83, 5.4) | 4.68 (2.83, 5.91) | 0.485 |
| D-Dimer | 1.65 (0.69, 3.96) | 1.1 (0.88, 3.24) | 1.21 (0.58, 3.95) | 3.4 (1.11, 4.05) | 0.497 |
| ALB | 29.43 ± 5.54 | 31.34 ± 3.87 | 30.84 ± 5.53 | 26.96 ± 5.42 | 0.014 |
| ALT | 37 (21, 74) | 30 (22.5, 56) | 50 (23, 220) | 29 (21, 65) | 0.172 |
| AST | 41 (28, 70) | 47 (39.5, 71.5) | 33 (24, 64) | 42 (30, 71) | 0.336 |
| TBIL | 12.3 (9, 18.1) | 12.3 (9.4, 16.85) | 12.3 (9.8, 18) | 12.5 (8.6, 18.1) | 0.989 |
| BUN | 8.8 (6.5, 12.7) | 9.7 (5.95, 13.2) | 8.7 (6.5, 10.2) | 8.4 (7.1, 12.7) | 0.887 |
| CRE | 72.8 (52.3, 106.9) | 76.2 (60.55, 99.55) | 72.8 (45.6, 109.6) | 68.6 (57.5, 92) | 0.908 |
| Glucose | 7.68 (6.6, 10.59) | 6.85 (5.77, 8.09) | 7.5 (6.6, 10.82) | 8.67 (7.04, 12.37) | 0.054 |
| Lactate | 3.3 (2.2, 4.4) | 1.71 (1.25, 2.85) | 2.9 (2.07, 3.7) | 4.3 (3.5, 4.9) | <0.001 |
| Septic shock |  |  |  |  | 0.835 |
| No | 42 (65) | 7 (64) | 20 (69) | 15 (60) |  |
| Yes | 23 (35) | 4 (36) | 9 (31) | 10 (40) |  |
| Mortality_7day, n (%) |  |  |  |  | 0.0206 |
| No | 54 (83) | 10 (91) | 26 (90) | 18 (72) |  |
| Yes | 11 (17) | 1 (9) | 3 (10) | 7 (28) |  |
| Mortality_28day, n (%) |  |  |  |  | <0.001 |
| No | 39 (60) | 11 (100) | 21 (72) | 7 (28) |  |
| Yes | 26 (40) | 0 (0) | 8 (28) | 18 (72) |  |
| ICU stay duration (days) | 10 (6, 17) | 7 (7, 10) | 15 (9, 23) | 6 (5, 11) | 0.004 |
| IMV_duration (hours) | 124 (73, 294) | 44 (11, 110) | 192 (120, 407.2) | 100 (68, 239.5) | 0.002 |

Abbreviations: WBC, white blood cells, SBP, systolic blood pressure, DBP, diastolic blood pressure, BUN,blood urea nitrogen, CRE, creatinine,

IMV, invasive mechanical ventilation.
